# Co-occurrence of Hyperacusis Accelerates With Tinnitus Burden Over Time and Requires Medical Care

**DOI:** 10.3389/fneur.2021.627522

**Published:** 2021-03-18

**Authors:** Fatma Refat, Jakob Wertz, Pauline Hinrichs, Uwe Klose, Hesham Samy, Rafeek Mohamed Abdelkader, Jörg Saemisch, Benedikt Hofmeier, Wibke Singer, Lukas Rüttiger, Marlies Knipper, Stephan Wolpert

**Affiliations:** ^1^Audio-Vestibular Unit, Department of Ear Nose Throat, Minia University, Minia, Egypt; ^2^Tübingen Hearing Research Centre, Department of Otolaryngology, Head and Neck Surgery, University of Tübingen, Tübingen, Germany; ^3^Department of Diagnostic and Interventional Neuroradiology, University of Tübingen, Tübingen, Germany

**Keywords:** tinnitus, hyperacusis, central gain, supra-threshold ABR wave, tinnitus duration

## Abstract

Although tinnitus represents a major global burden, no causal therapy has yet been established. Ongoing controversies about the neuronal pathophysiology of tinnitus hamper efforts in developing advanced therapies. Hypothesizing that the unnoticed co-occurrence of hyperacusis and differences in the duration of tinnitus may possibly differentially influence the neural correlate of tinnitus, we analyzed 33 tinnitus patients without (T-group) and 20 tinnitus patients with hyperacusis (TH-group). We found crucial differences between the T-group and the TH-group in the increase of annoyance, complaints, tinnitus loudness, and central neural gain as a function of tinnitus duration. Hearing thresholds did not differ between T-group and TH-group. In the TH-group, the tinnitus complaints (total tinnitus score) were significantly greater from early on and the tinnitus intensity distinctly increased over time from ca. 12 to 17 dB when tinnitus persisted more than 5 years, while annoyance responses to normal sound remained nearly constant. In contrast, in the T-group tinnitus complaints remained constant, although the tinnitus intensity declined over time from ca. 27 down to 15 dB beyond 5 years of tinnitus persistence. This was explained through a gradually increased annoyance to normal sound over time, shown by a hyperacusis questionnaire. Parallel a shift from a mainly unilateral (only 17% bilateral) to a completely bilateral (100%) tinnitus percept occurred in the T-group, while bilateral tinnitus dominated in the TH-group from the start (75%). Over time in the T-group, ABR wave V amplitudes (and V/I ratios) remained reduced and delayed. By contrast, in the TH-group especially the ABR wave III and V (and III/I ratio) continued to be enhanced and shortened in response to high-level sound stimuli. Interestingly, in line with signs of an increased co-occurrence of hyperacusis in the T-group over time, ABR wave III also slightly increased in the T-group. The findings disclose an undiagnosed co-occurrence of hyperacusis in tinnitus patients as a main cause of distress and the cause of complaints about tinnitus over time. To achieve urgently needed and personalized therapies, possibly using the objective tools offered here, a systematic sub-classification of tinnitus and the co-occurrence of hyperacusis is recommended.

## Introduction

Tinnitus and hyperacusis are among the most frequent audiological disorders and have a significant socioeconomic impact on the health care system (1). In subjective tinnitus, by far the most common form, a phantom noise is perceived without any external stimulus, whereas in hyperacusis, sounds are perceived as annoying even at low sound levels (2, 3). The prevalence of hyperacusis in adults is about 9%, with a much higher estimated number of unreported cases (4, 5). In hyperacusis patients, the incidence of tinnitus may be even higher compared to individuals without hyperacusis (6). According to the literature, 80 to 90% of patients with hyperacusis have additional tinnitus, while only 30 to 50% of tinnitus patients have additional hyperacusis (2, 7–11). This suggests that hyperacusis is frequently associated with tinnitus, whereas tinnitus is more common without the co-occurrence of hyperacusis.

Although tinnitus represents a major global burden, no causal therapy has yet been established. One reason for this is ongoing controversy about the neuronal pathophysiology of tinnitus. While many authors postulate that peripheral auditory damage leads to tinnitus by an increase in neural gain (12–22), recent studies propose that tinnitus without hyperacusis occurs when the reduced auditory input fails to increase neural gain (23–25).

A particular challenge for clinical studies is that tinnitus and hyperacusis are subjective phenomena, for which objective measurements are still missing (26). Factors such as hearing loss or hyperacusis were suggested to contribute to misinterpretations, due to overlapping effects (27). Newer studies consistently matched patients and control subjects to their hearing threshold, or included only patients with normal hearing and excluded patients with the co-occurrence of hyperacusis (23, 25, 28).

A hitherto, largely unexplored, factor is the duration of chronic tinnitus. In three recent publications, the transition from acute to chronic tinnitus was examined (28–30). One study included normal hearing subjects with tinnitus lasting longer than 6 months (28) and classified patients into 3 categories according to the tinnitus duration: (i) acute, < 1 month (ii) chronic, > 6 months, (iii) subacute, between 1 and 6 months. In the subacute stage of tinnitus, a decreased amplitude and prolonged latency of wave V of the auditory brainstem response (ABR) were observed (28), confirming previous studies that excluded tinnitus patients with a co-occurrence of hyperacusis (23). Another study, using fluorodeoxyglucose — Positron Emission Tomography (FDG-PET), examined time-dependent changes in chronic tinnitus and found a correlation between tinnitus duration and brain metabolism in the right inferior frontal, right ventro-medial prefrontal, and right posterior cingulate cortex (31). To the best of our knowledge, there are no studies that compared psychoacoustic characteristics with the fine structure of ABR over time (years) in tinnitus patients with and without hyperacusis.

The aim of the present study was to investigate functional changes depending on tinnitus duration and the presence of hyperacusis in patients with chronic tinnitus. Control subjects and tinnitus patients with and without the co-occurrence of hyperacusis were recruited and divided into four groups according to the duration of their complaints.

## Materials and Methods

The study was approved by the ethics committee of Tübingen University (faculty of medicine) (ethical approval-number 264-2016BO1 as well as follow up study 391/2018BO2). Written informed consent was obtained from all participants. All methods were used according to the Declaration of Helsinki' by the World Medical Association (WMA) for human research ethics.

### Participants

**Figure 1:**
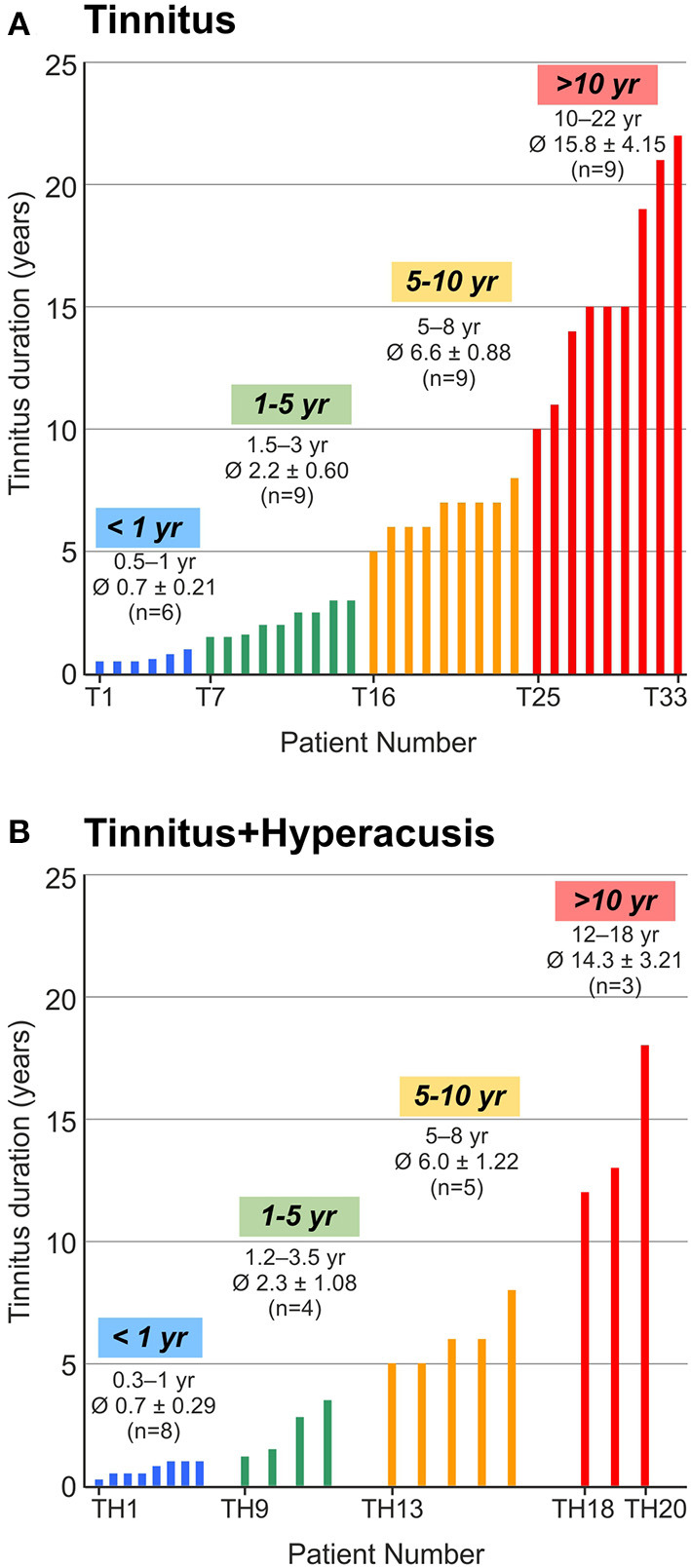
Distribution of participants according to the time of persistence of their tinnitus (tinnitus duration in years, yr). **(A)** 33 participants with tinnitus (T1 to T33) were classified into 4 groups of tinnitus duration up to 1 year (< 1 yr), more than 1 year up to 4.9 years (1–5 yr), from 5 years up to 9.9 years (5–10 yr) and from 10 years and more (> 10 yr). **(B)** 20 participants with tinnitus and hyperacusis (TH1 to TH20) were classified accordingly. Groups were color coded by blue, green, yellow, and red in respect to tinnitus duration throughout this manuscript. Calculation with Kruskal-Wallis test showed no difference between tinnitus and tinnitus+hyperacusis within the tinnitus duration groups.

Out of 96 subjects 43 could be assigned to the control group (age: 26.5 ± 5.8 years, between 18 - 45 years, 20 men and 23 women) and 53 to the tinnitus group. Among these, 33 patients complained of tinnitus without co-occurrence of hyperacusis (age: 32.6 ± 11.5 years, between 20 and 61 years, 22 men and 11 women, “T-group”, Figure 1A and Supplementary Tables 1, 2), and 20 patients complained of tinnitus with co-occurrence of hyperacusis (age: 26.9 ± 6.9 years, between 18 and 49 years, 6 men and 14 women, “TH-group”, Figure 1B and Supplementary Tables 1, 2). According to inclusion criteria, pure tone audiometry hearing thresholds did not exceed 20 dB at each frequency from 0.125 to 3 kHz and did not exceed 50 dB at each frequency from 4 to 10 kHz in addition (Study exclusion criteria Supplementary Table 3). All participants were then classified according to their tinnitus duration into four groups (Figure 1): (I) tinnitus duration less than 1 year ( ≤ 1 year, yr), (II) 1 year up to 5 years (1–5 yr), (III) 5 years up to 10 years (5–10 yr), (IV) tinnitus for more than 10 years (> 10 yr).

### Hyperacusis Questionnaire

In order to assess the presence of hyperacusis and to differentiate it from phonophobia, the Hyperacusis Questionnaire (Hyperakusis-Inventar, HKI) was administered to all participants (32, 33). All participants had to answer 9 statements in the questionnaire, and were asked to rank this statement as: always true, often true, sometimes true, or not true (evaluated with a score from 3 to 0). The questionnaire results were calculated according to the answers of participants and are scored from 0 to 27. Participants were considered having hyperacusis if the score was above 11 (32).

### Tinnitus Questionnaire

The German Goebel-Hiller-Score (G-H-S) tinnitus questionnaire was used to assess different aspects concerning tinnitus severity, laterality, emotional distress, cognitive distress, annoyance in the last days, penetrance, auditory perceptual difficulty, sleep disturbances and somatic complaints scores (34). The patients had to answer 52 statements in the questionnaire, and were asked to rank this statement as: true, partly true, or not true. The different questionnaire scores were collected, and each sub-score was calculated according to the patients' responses.

### Audiological Evaluation

The ear examination was carried out by ENT physicians from the Department of Otolaryngology, Head and Neck Surgery at the University of Tübingen.

Tympanometry and acoustic reflex measurements for 0.5, 1, 2, and 4 kHz and for 80 to 100 dB sound pressure level (SPL) were performed with a Madsen-Zodiac 901 (GN Otometrics, Münster, Germany) to ensure normal middle ear transmission.

Hearing thresholds were determined by pure tone audiometry with the AT 900 Audiometer (Auritec, Medizindiagnostische Systeme GmbH, Hamburg, Germany) from 0.125 to 10 kHz (0.125/0.25/0.5/1/1.5/2/3/4/6/8 and 10 kHz). Speech audiometry was measured by the German monosyllabic “Freiburger” Test.

For all tinnitus patients and all tinnitus patients with co-occurrence of hyperacusis, sound of different frequencies and intensities were presented to the individual ears to identify tone pitch and loudness closest to the perceived tinnitus.

The ABR testing was done by using the system GSI Audera (Grason-Stadler, Eden Prairie, USA) device, with Telephonics TDH 39p headphones (Telephonics, Farmingdale, USA). Measurements were performed with two-channel recording using four electrodes (Neuroline 720, Ambu, Bad Nauheim, Germany) at predetermined positions according to the International Electrode System 1020 standard system after cleaning the patients' skin with abrasive paste (Nuprep Skin Prep Gel, Weaver and Company, Aurora, USA) and attaching the electrodes with electrolytic paste and adhesive tape. The predetermined positions were active electrodes at the left mastoid (M1) and at the right mastoid (M2), a ground electrode at the forehead near the eyebrows, and a reference electrode centered close to the hairline as described in the manual of the above-mentioned device. The electrodes were connected to the pre-amplifier. The ABR was recorded ipsi-lateral in response to broadband acoustic click stimuli (0.1 ms duration) presented at 25 to 75 dB normalized hearing level (nHL) in 10 dB steps. The click was presented at repetition rate of 11.1 Hz with 2,000 repetitions. ABR signals were bandpass filtered between 150 and 3,000 Hz and recorded for 10 ms. Electrode impedance was maintained at <5 kΩ and not more than 2 kΩ impedance difference between two single electrodes.

### Calculation of Supra-Threshold ABR Wave Fine Structure

From the ABR response of individual ears at a given stimulation level, positive and subsequent negative voltage deflection maxima and minima (peaks) were specified within pre-defined time interval of between 1–2, 3–4, 5–6, and 6–7 ms after stimulus onset. Within the consecutive time intervals, ABR wave I, III, V, and VI amplitudes were determined as the peak-to-peak voltage difference between positive and negative peak. ABR wave latencies were defined as the time point of the positive peak from the respective wave. Amplitude and latency data were manually extracted from the output of the ABR function and verified by an expert audiologist. Data from individual supra-threshold ABR wave amplitudes, wave latencies, and inter-peak latencies were analyzed for individual ears before the averaged data from both ears were used to calculate the group mean for each experimental group (T and TH, < 1 year, 1–5 years, 5–10 years, > 10 years).

### Statistical Analysis

#### Statistical Tests

Grouped data were tested for normality by the Shapiro-Wilk Test and for equality of distribution with Levene Test. To test for the significance of group differences Kruskal-Wallis Test for non-normal distributed data was applied. ABR wave amplitudes and latencies were compared by 2-way ANOVA for the factors study group differences, stimulus level, and interaction. Resulting *P*-values smaller than the criterion of α = 0.05 were considered as statistically significant. Due to the small sample sizes resulting from the subdivision into eight experimental groups we did not determine the statistical power (β). The correlation of two measurement parameters was verified upon the Pearson Correlation Momentum.

#### Data Distributions

Data from Hyperacusis (HKI) and Tinnitus Questionnaires, tinnitus intensity and frequency matching procedures, and Wave V/I and III/I ratios failed normal distribution. Though, distributions were not statistically significant different between study groups.

#### Data Presentation

If not otherwise indicated data are presented as group mean and standard deviation (SD) for the number of subjects or ears (n) specified in the figure legends.

## Results

Toward the aim of identifying characteristics of auditory function that may differ in tinnitus groups with or without hyperacusis over time, we included 96 subjects, who were subclassified using the hyperacusis questionnaire (HKI) developed by Goebel und Berthold (32) and the (G-H-S) tinnitus questionnaire (34). In sum, 33 patients complained of tinnitus without the co-occurrence of hyperacusis (“T-group”) (Figure 1A and Supplementary Table 1A), and 20 patients complained of tinnitus with hyperacusis (“TH-group”) (Figure 1B and Supplementary Table 1A). In detail, the distribution of tinnitus duration (Td) was:

**T-group:** 6 patients with Td up to 1 year (Td = 0.5–1; mean age: 35.2); 9 patients 1–5 years (Td = 1.5–3; mean age = 34.1); 9 patients 5–10 years (Td = 5–8; mean age = 29), and 9 patients with duration above 10 years (Td = 10–22; mean age = 33.1) (Figure 1 and Supplementary Table 1A).

**TH-group:** 8 patients with Td up to 1 year (Td = 0.5–1; mean age = 29); 4 patients 1–5 years (Td = 0.25–1; mean age = 24.2); 5 patients 5–10 years (Td = 1.2–3.5; mean age = 27), and 3 patients with duration above 10 years (Td = 12–18; mean age = 25).

### Hearing Thresholds Do Not Differ Between T- and TH-Groups

**Figure 2:**
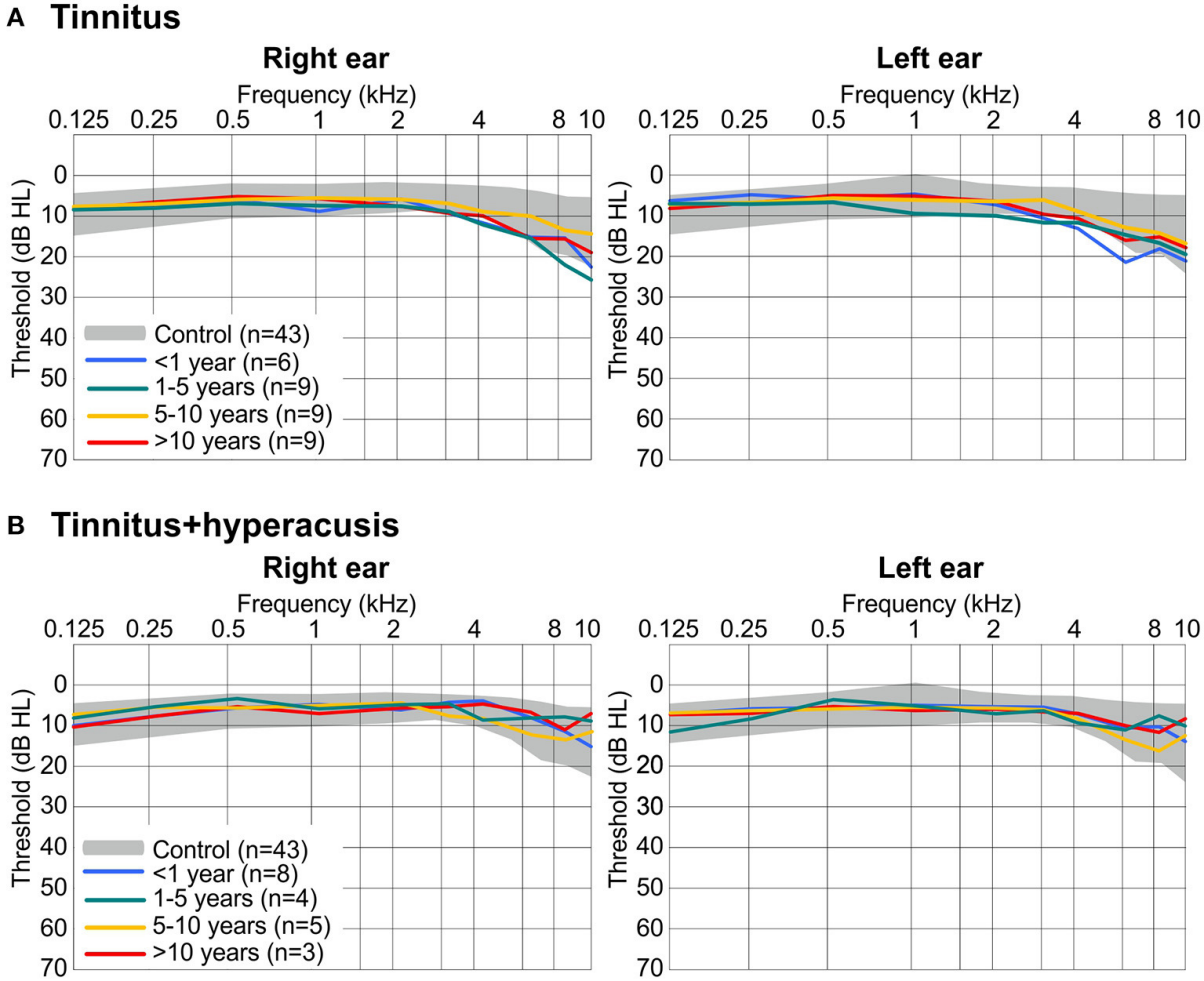
Hearing sensitivity for right ear (left) and left ear (right) determined by pure tone audiometry (threshold in dB hearing level, HL). **(A)** Thresholds range from 43 controls (gray shaded areas display the normal range of ± one standard deviation) and mean of 33 participants with tinnitus only, split into 4 groups according to the duration of tinnitus. **(B)** Mean thresholds from 20 participants with tinnitus + hyperacusis, split into 4 groups according to the duration of their tinnitus. The same values for controls are displayed in **(A,B)**. Hearing thresholds did not statistically significant differ in ears or in participant groups using Kruskal-Wallis test with *P* > 0.05.

To investigate whether tinnitus groups with and without hyperacusis can be distinguished on the level of their hearing thresholds, we assessed the hearing function by a pure tone audiogram as described in the methods. We found no statistically significant differences in hearing thresholds between 0.125 and 10 kHz for either ear between the four tinnitus-duration groups without the co-occurrence of hyperacusis (Figure 2A) or with the co-occurrence of hyperacusis (Figure 2B). This indicates that age-dependent hearing thresholds do not differ between the groups.

### Tinnitus Loudness Intensity Decreased Over Time in the T-Group, but Increased in the TH-Group

**Figure 3:**
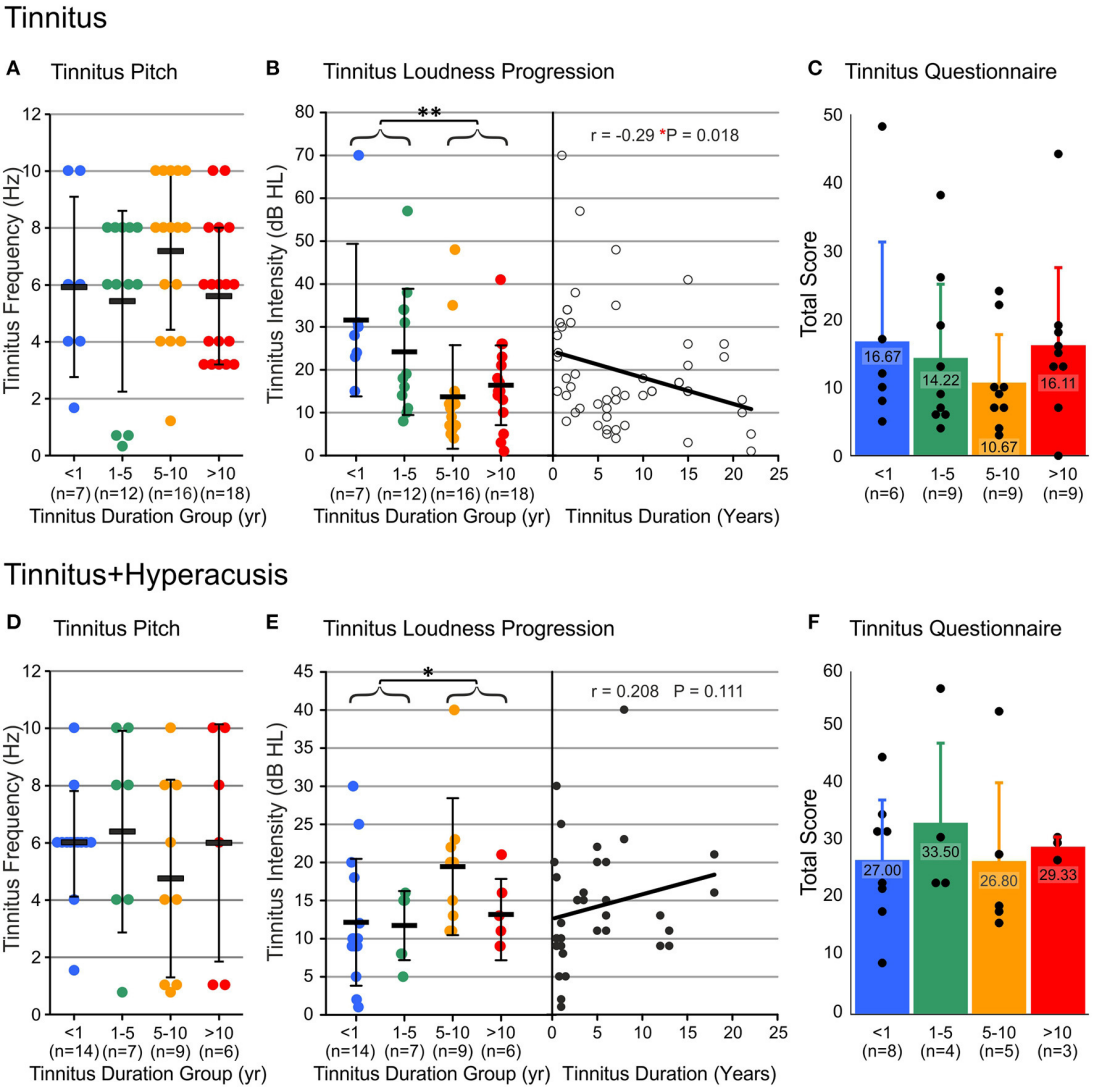
Tinnitus percept significantly changes over tinnitus duration in tinnitus and tinnitus+hyperacusis groups. **(A**, **D)** Subjectively matched percept of tinnitus pitch was stable along the time of tinnitus duration in both, tinnitus (*P* = 0.070) and tinnitus+hyperacusis (*P* = 0.534) participants, calculated with Kruskal-Wallis test. **(B**, **E)** The loudness of the tinnitus percept, determined by the intensity of the matching tone decreased over time of tinnitus duration in the tinnitus only group with a statistically significant drop around 5 years (**B**, *P* = 0.005 for < 5 years and > 5 years). In tinnitus+hyperacusis participants, the tinnitus was significantly louder after > 5 years of tinnitus duration, when no more participants with very moderate tinnitus percept could be found (**E**, *P* = 0.015 for < 5 years and > 5 years). Level of significance was calculated with Mann-Whitney U-test is illustrated in the figures (*P ≤ 0.05; **P < 0.01). A linear trend for the progression of tinnitus loudness over the duration of tinnitus in single ears of the tinnitus group **(B)** and the tinnitus+hyperacusis group **(E)** reached statistical significance in the tinnitus group (**B**, *P* = 0.018, *n* = 52 ears) but not in the tinnitus+hyperacusis participants (**E**, *P* = 0.111, *n* = 36 ears), for whom the opposite of the trend visible in the tinnitus group was found. **(C,F)** Total score from Goebel-Hiller Tinnitus Questionnaire indicated a higher affection from tinnitus in the tinnitus+hyperacusis participants than in the participants with only tinnitus (all tinnitus vs. all tinnitus+hyperacusis participants: *P* < 0.001 using Mann-Whitney *U*-test, and 2-way ANOVA). Calculation with Kruskal-Wallis test showed no difference between tinnitus and tinnitus+hyperacusis within the tinnitus duration groups. Data show mean ± SD. Single dots show number of ears.

To get more detailed information whether the tinnitus percept differs between groups, we analyzed tinnitus frequency, tinnitus intensity, and tinnitus score (34) between the four tinnitus-duration groups for both the T-group and the TH-group (Figure 3).

The frequency of the tinnitus percept did not significantly change with tinnitus duration in the T-group (Figure 3A) or the TH-group (Figure 3D), and it did not differ between the groups, resulting in an averaged tinnitus frequency for the T-group < 1 year (5.93 ± 3.17 kHz), 1–5 years (5.43 ± 3.18 kHz), 5–10 years (7.19 ± 2.76 kHz), > 10 years (5.61 ± 2.4 kHz), and the TH-group < 1 year (5.96 ± 1.84 kHz), 1–5 years (6.39 ± 3.52 kHz), 5–10 years (4.75 ± 3.45 kHz), > 10 years (6 ± 4.15 kHz).

The subjective tinnitus level (loudness) decreased over time in the T-group (Figure 3B), but was significantly greater for a tinnitus duration of < 5 years than for tinnitus durations longer than 5 years (< 5 years, 26.9 ± 15.8 dB, n = 19; > 5 years, 16.1 ± 11.7 dB, n = 34; ^**^P = 0.005, Figure 3B) time. In contrast, in the TH-group (Figure 3E), the tinnitus intensity increased with tinnitus duration (< 5 years, 12 ± 7.2 dB, n = 21; > 5 years, 16.9 ± 8 dB, n = 15; ^*^P = 0.015, Figure 3E). To see the continuous change of the tinnitus loudness with time, the tinnitus loudness matching (tinnitus level dB HL) in the T-group (Figure 3B, right) and TH-group (Figure 3E, right) were plotted as a function of tinnitus duration between 3 months and 22 years. Linear regression disclosed a decrease in tinnitus loudness for tinnitus patients (^*^P = 0.018, Figure 3B, right). No significant increase for tinnitus patients with the co-occurrence of hyperacusis was found (*P* = 0.111, Figure 3E, right).

### Tinnitus Burden Remained Constant Over Time but Accelerated Significantly With the Co-occurrence of Hyperacusis

The evaluation of the tinnitus G-H-S in the different groups showed no significant change in total score (emotional, distress) over time, not for the T-group (Figure 3C) nor the TH-group (Figure 3F). Surprisingly, even though the perceived tinnitus level from the tinnitus loudness matching was higher in the T-group as compared to the TH-group (Figures 3B,E), the overall score thresholds were significantly higher in the TH-group than in the T-group (Figures 3C,F, *p* < 0.001), indicating that total G-H-S tinnitus score thresholds are different in these groups from the acute stage onwards.

**Figure 4:**
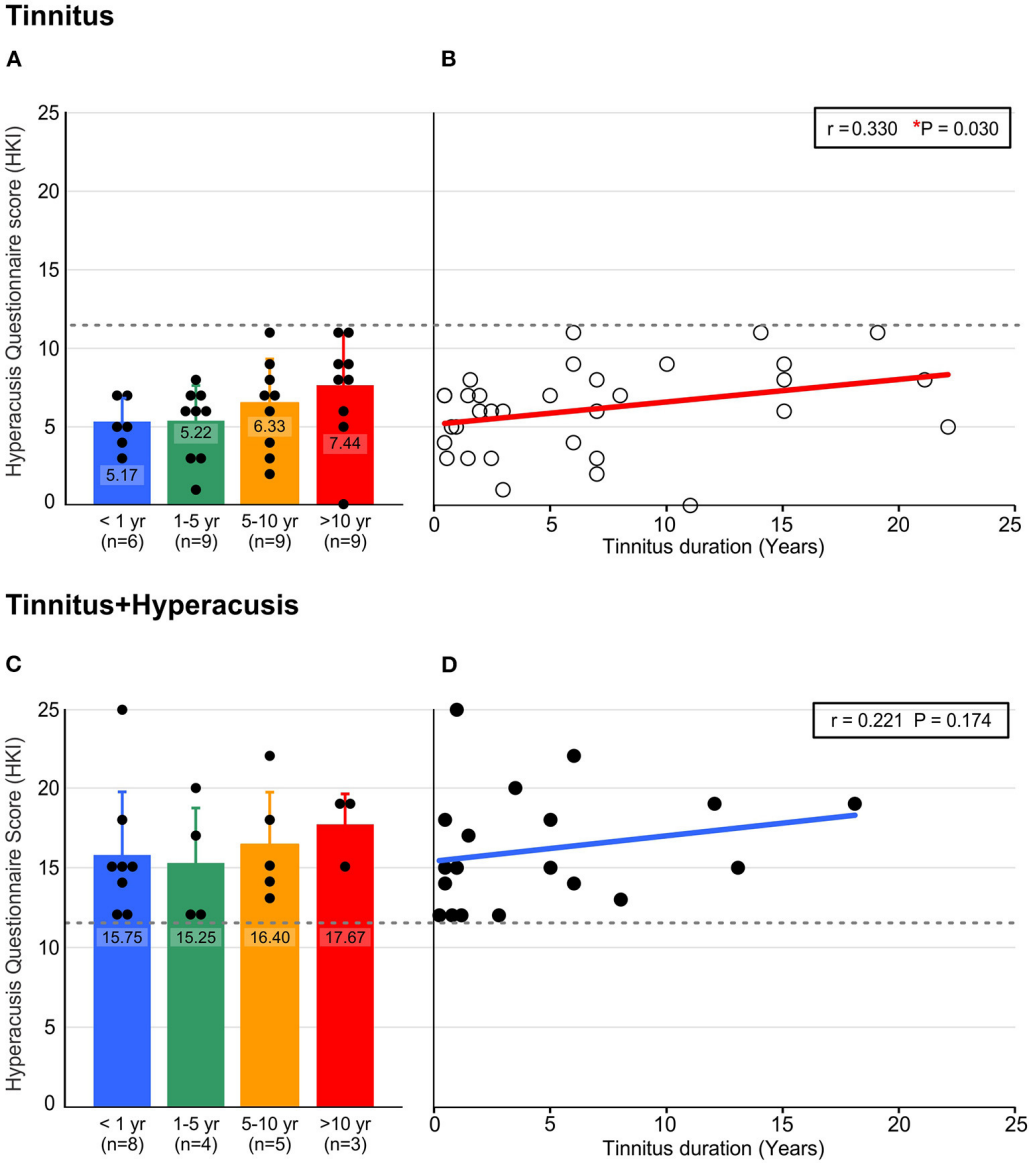
Hyperacusis percept significantly increases in the participants with tinnitus with the longer persistence of their tinnitus (duration in years), determined by Hyperacusis Questionnaire score (HKI). **(A)** Tinnitus participants classified according to their tinnitus duration did not reach the criterion for being classified as hyperacusis suffering (HKI < 12), but reached higher scores with the longer persistence of their tinnitus. **(B)** Linear trend over time of tinnitus duration suggests a significant increase of affection by hyperacusis with tinnitus duration (*P* = 0.03, *n* = 33). **(C)** Participants with tinnitus and classified as hyperacusis sufferers (HKI ≥ 12) experienced continuing hyperacusis over the duration of their tinnitus. **(D)** The hyperacusis in the tinnitus+hyperacusis participants followed a trend of hyperacusis reinforcement with tinnitus duration, though this was not statistically significant (*P* = 0.174, *n* = 20). Data in A, C show mean ± SD. r correlation coefficient, *P < 0.05, HKI; Hyperacusis Inventory.

This may be related to the group differences we observed when the total score of the hyperacusis questionnaire (HKI) of Goebel und Berthold (32) was identified between the groups, depending on the tinnitus duration. Thus, in the T-group (Figures 4A,B), but not in the TH-group (Figures 4C,D), an increase over time in the burden and complaints through hyperacusis was identified (^*^P = 0.030). This suggested that the probability of co-occurrence of some characteristics of hyperacusis may increase with time.

### Over Time, the Tinnitus Percept Shifts From Unilateral to Bilateral in the T-Group but Is Prevalently Bilateral in TH-Group From Early On

**Figure 5:**
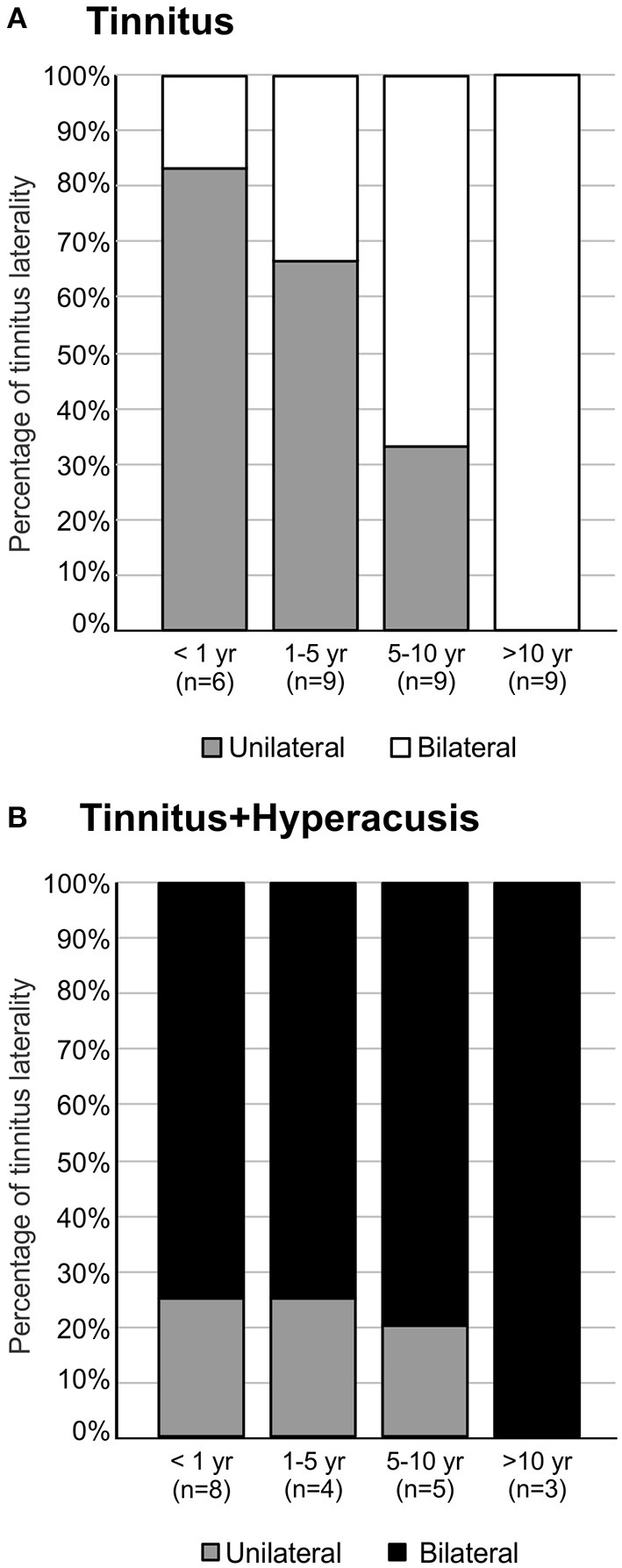
Tinnitus laterality ends in bilateral percepts with time of persistence (duration > 10 years). **(A)** The majority of participants with tinnitus experience tinnitus unilateral if tinnitus duration was < 5 years. Above 5 years, most participants experienced bilateral tinnitus (Chi-Square Test, *P* = 0.004). **(B)** Participants with tinnitus+hyperacusis experienced more bilateral tinnitus independent of tinnitus duration, ending in complete bilaterality of tinnitus for durations longer than 10 years (Chi-Square Test, *P* = 0.816).

Not only does the probability of hyperacusis increase over time, the probability of tinnitus in both ears also significantly increased over time. Thus, in the T-group, the tinnitus percept was mostly unilateral in individuals with a short duration of tinnitus < 1 year (Figure 5A). Over the duration of tinnitus, however, the tinnitus percept shifted to a completely bilateral sensation, (^**^P = 0.002). In contrast, in the TH-group, a bilateral experience of tinnitus dominated from the beginning, with a stable proportion up to 10 years, (*P* = 0.816, Figure 5B). All patients with a tinnitus experience for more than 10 years experienced bilateral tinnitus, irrespective of concomitant hyperacusis.

The results suggest that the majority of tinnitus patients undergo a change from unilateral to bilateral tinnitus, and that this process takes a maximum of 10 years, whereas patients with tinnitus and hyperacusis usually have bilateral tinnitus from very early stages.

### ABR Wave V Remains Reduced and Delayed in T-Group, and ABR Wave III and V Remain Enhanced and Shortened in TH-Group Over Time

**Figure 6:**
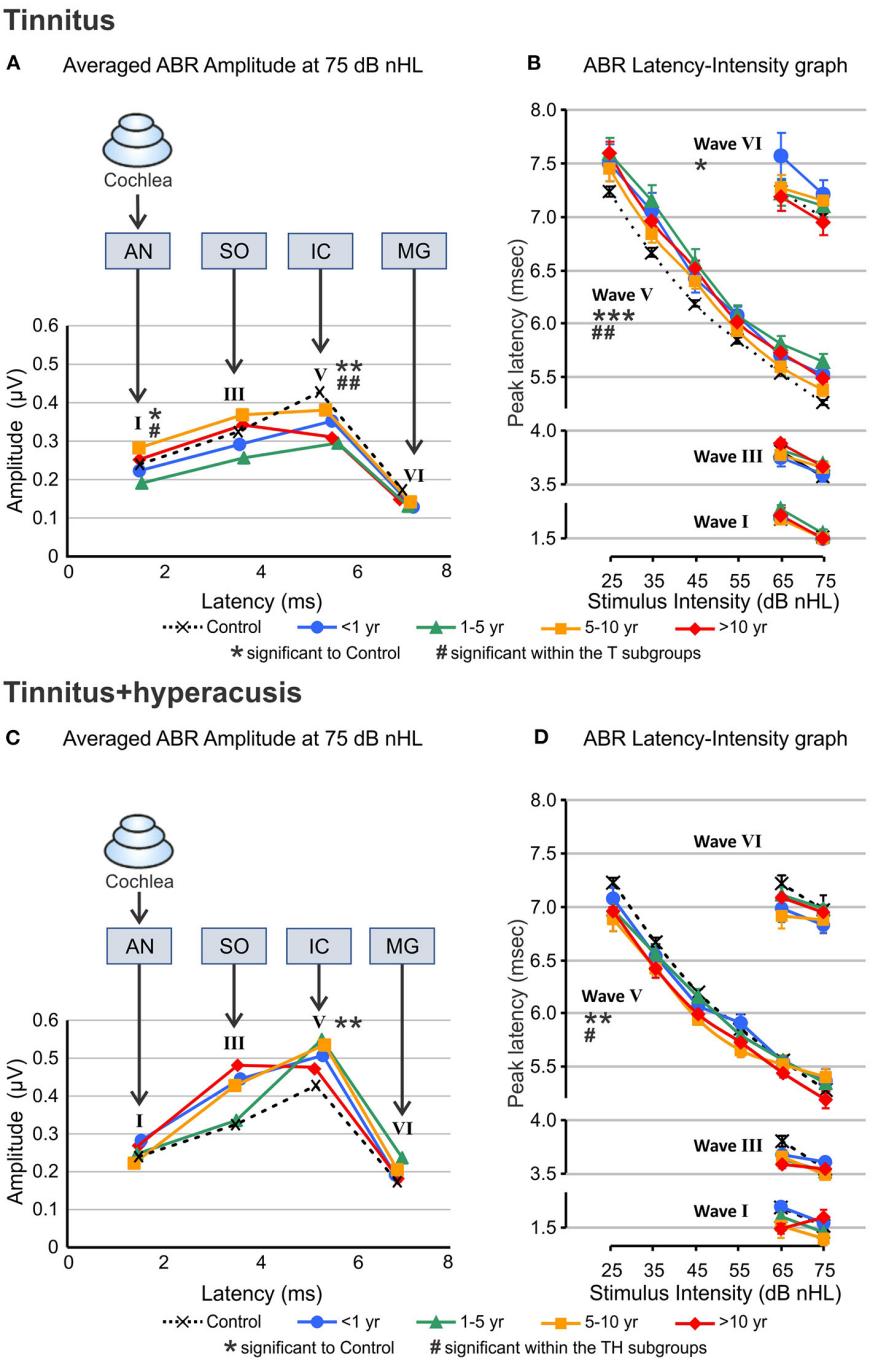
Auditory brainstem response wave amplitudes and latencies calculated from ABR responses to 25–75 dB nHL click stimuli. **(A**, **C)** Average amplitude of ABR wave I, III, V, and VI for 75 dB nHL stimuli for participants with tinnitus **(A)** and both, tinnitus+hyperacusis **(C)** categorized according to the duration of their tinnitus (colored curves) and compared to healthy controls (black dashed line and symbols). The amplitudes for either wave were displaced horizontally according to the average latency of the respective wave (latency, ms). AN, SO, IC, and MG illustrate the presumed brain structure origin for the wave I, II, V, and VI, respectively. **(B, D)** Peak latency as a function of stimulus intensity for the ABR Wave I, II, V, and VI across the stimulation range from 25 to 75 dB nHL. Peak values were clearly measurable only from 65 to 75 dB nHL. Data represent responses from both ears averaged of *n* = 33 tinnitus, *n* = 20 tinnitus+hyperacusis, and *n* = 43 control participants. Wave V exhibit reduced amplitude and prolonged latency regardless of the duration of the tinnitus in T-group, while in TH-group the increase in the amplitude become more over time. Level of significance is illustrated in the figures (significance to control: *P ≤ 0.05; **P < 0.01; ***P < 0.001; significance within the subgroups: ^#^*P* ≤ 0.05; ^*##*^*P* < 0.01). For detailed statistics (2-way ANOVA) see Supplementary Table 4. ABR, auditory brainstem response; dB, decibel; nHL, normalized hearing level; AN, auditory nerve; SO, superior olivary complex; IC, inferior colliculus; MG, medial geniculate body of the thalamus.

As a most striking overall finding, the wave I (F_(4, 285)_ = 2.481, ^*^P = 0.0441) and V (F_(4, 869)_ = 3.653, ^**^P = 0.0058) amplitudes were smaller (Figure 6A) and the wave V latency prolonged (F_(4, 869)_ = 16.28, ^****^P < 0.0001, Figure 6B) in the T-group compared to controls, but wave III and V (F_(4, 704)_ = 3.698, ^**^P = 0.0055) was larger (Figure 6C) and shortened (F_(4, 704)_ = 3.698, ^**^P = 0.0055, Figure 6D) in the TH-group (Supplementary Table 4). This occurs with large variations and no clearly gradual changes dependent on tinnitus duration. A few interesting observations on the differences in ABR wave amplitudes and in tinnitus duration are, however, worth mentioning. Although no statistically significant differences for wave I amplitudes could be detected in the TH-group (Figure 6C and Supplementary Table 4) as a function of the years of tinnitus duration, it was noticed that in the T-group a tinnitus persistence of < 5 years was accompanied by reduced amplitudes in wave I, while a tinnitus duration over 5 years lead to ABR wave I that was slightly higher than in control subjects. The same tendency was noted for wave III, however not statistically significant. This suggests that over time, lower auditory brain regions (ABR wave I to III) may start to generate gain related to the increased probability of the co-occurrence of hyperacusis.

Peak latencies of ABR wave I, III, and VI were measured for 65 and 75 dB nHL stimuli, and of ABR wave V for 25–75 dB nHL stimulus levels (Figures 6B,D). In the T-group, particularly for latencies of ABR wave V for all stimulus levels (F_(4, 869)_ = 16.28, ^****^P < 0.0001, Figure 6B), but also with variations dependent on stimulus level for other ABR waves, delayed ABR wave peaks were observed. This trend appeared to be most pronounced for tinnitus durations < 5 years and for higher sound stimulus levels (F_(3, 370)_ = 4.674, ^**^P = 0.0032, Figure 6B). In contrast, wave V latencies in the TH-group were shorter (F_(4, 704)_ = 3.698, ^**^P = 0.0055), especially at the low stimulus levels, and with a trend such that this feature increased with the duration of tinnitus (F_(3, 205)_ = 3.488, ^*^P = 0.0167, Figure 6D).

### Central Output to Reduced Auditory Input (Neural Gain) Remained Reduced Over the Years in the T-Group and Enhanced in TH-Group

**Figure 7:**
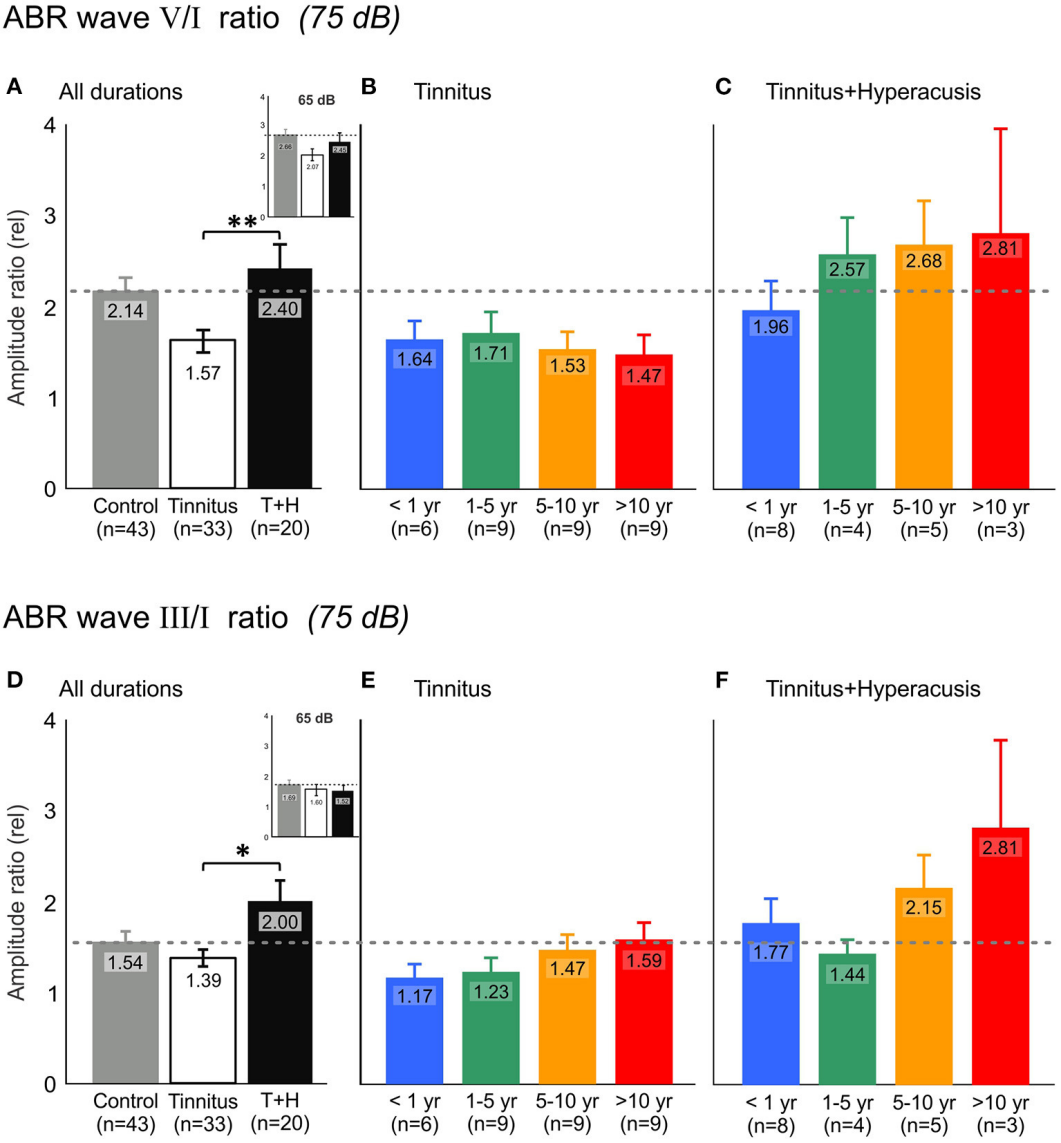
Auditory brainstem response (ABR) wave V and wave I relation (V/I ratio) demonstrates increased auditory response gain in tinnitus+hyperacusis participants but not in participants with tinnitus only. **(A)** ABR wave V/I amplitude ratio averaged for all participants of tinnitus (open bar) and tinnitus+hyperacusis (black bar) participants for 75 dB nHL click stimulus evoked ABR responses compared to healthy controls (gray bar). The inserts show the result at 65 dB nHL. Higher values illustrate a larger auditory output/input relation, that is, a higher efficiency of the auditory input (wave I, generated in the auditory nerve) to generate a larger wave amplitude within the auditory midbrain (wave V, refers to inferior colliculus), referred to as auditory central gain. **(B,C)** ABR wave V/I ratio for participants with tinnitus and tinnitus+hyperacusis, respectively, categorized according to the time of persistence of their tinnitus (tinnitus duration). The horizontal dashed line marks control level. **(D)** ABR wave III/I ratio illustrating the efficiency of the auditory input (wave I, generated in the auditory nerve) to generate a larger wave amplitude within the auditory brainstem (wave III, refers to the superior olivary complex), referred to as auditory brainstem gain. **(E,F)** ABR wave III/I ratio for participants with tinnitus and tinnitus+hyperacusis, respectively, categorized according to their tinnitus duration. At 75 dB nHL T, TH: V/I: *P* = 0.001, III/I: *P* = 0.026, 65 dB nHL T, TH: V/I: *P* = 0.286, III/I: *P* = 0.958 using Mann-Whitney *U*-test. At 75 dB nHL T: V/I: *P* = 0.733, T: III/I: *P* = 0.430, TH: V/I: *P* = 0.613, TH: III/I: *P* = 0.596 using Kruskal-Wallis test. Data represent mean ± standard error (s.e.m.). Level of significance is illustrated in the figures (*P ≤ 0.05; **P < 0.01).

ABR wave ratio V/I at 75 dB nHL (Figures 7A–C) and 65 dB nHL (Figure 7A insert), and ABR wave ratio III/I at 75 dB nHL (Figures 7D–F) and 65 dB nHL (Figure 7D insert) where either averaged for both T-group and TH-group for all tinnitus durations (Figures 7A,D) or compared for the individual tinnitus durations for the T-group (Figures 7B,E) and TH-group (Figures 7C,F).

In comparison to control subjects (dotted line), a strikingly reduced ABR wave V/I ratio (Figure 7A) occurred in the T-group. In the TH-group, an enhanced ABR wave III/I (^*^P = 0.026, Figure 7D) and V/I ratio (^**^P = 0.001, Figure 7A) was observed for an average of the tinnitus duration groups at 75 dB nHL, but not at 65 dB nHL (Figures 7A,D insert), suggesting that changes in brainstem gain of TH-groups occurs only at higher simulation levels. With increasing tinnitus duration, the ABR wave V/I ratio in the T-group tended to decrease (Figure 7B), while particularly the ABR wave III/I ratio in the TH-group tended to increase over the years of tinnitus duration (Figure 7F). Interestingly, as evident through ABR wave III changes over time in the T-group (Figure 6A), the ABR wave III/I ratio also tended to increase in the T-group over the duration of tinnitus (Figure 7E).

In summary, neural central gain (ABR wave V/I ratio) decreased in the T-group, while ABR wave III/I and ABR wave V/I ratio increased in the TH-group over the duration of tinnitus. In the T-group, not only did over time the ABR wave V/I ratio tend to be more reduced, but ABR wave III/I ratio tended to be less diminished, challenging the concept of an increase in hyperacusis characteristics in tinnitus patients over the time of tinnitus duration.

## Discussion

In the present study, tinnitus patients with and without the co-occurrence of hyperacusis were grouped according to the duration of their tinnitus to determine time-dependent changes in objective and subjective hearing assessments. We identified significant differences between the T-group and TH-group in tinnitus loudness and burden, but also in central output auditory responses to reduced auditory input as a function of time that explain previous controversial views of the neural correlate of tinnitus as partially un-diagnosed co-occurrence of hyperacusis.

### Hearing Thresholds and Tinnitus Frequency Do Not Differ Between T- and TH-Groups

Although there is no definite criterion for classifying tinnitus by duration, several studies agreed in suggesting that tinnitus with a duration shorter than 1 month be classified as acute, while longer than 6 months of tinnitus would be classified as chronic (28, 29, 35). Thus, the four subgroups selected in the present study would all have to be classified as sub-entity-specific differences of chronic tinnitus.

The tinnitus frequency revealed as a relatively constant and robust factor. Both the duration and the presence of hyperacusis had no influence on the pitch of the tinnitus (Figures 3A,D), at least in our group that included patients with normal hearing thresholds or mostly only mild hearing loss (> 40 dB HL). Importantly, in our study hearing thresholds did not differ between groups or over the duration of tinnitus (Figure 2).

As hearing thresholds in cochlear, sensory-neural hearing loss can mainly be attributed to the electromechanical properties of outer hair cells (OHCs) (36, 37), and previous studies find no difference in hearing threshold or electromechanical properties comparing tinnitus to control groups (36–41), or hyperacusis (5, 42, 43), it was suggested that neither tinnitus nor hyperacusis may be causally linked to the loss of OHCs. Hearing loss was, however, classified as a profound risk factor for tinnitus (10, 41, 43). Indeed, if the loss of hearing thresholds would causally impact the perceived tinnitus frequency, we might expect that with time, the tinnitus frequency would be shifted to lower frequencies, as does the hearing loss in presbycusis subjects (44, 45), a feature that up to now was not reported. The recruitment conditions of the present study, that restricted the participants to those with a mild hearing loss only may be a limitation, however. A later confirmation of the here identified functional biomarker differences for T-group and TH-group may be required in subjects with profound hearing loss.

### Tinnitus Loudness Levels Decreased Over Time in the T-Group, but Increased in the TH-Group

We observed tinnitus loudness and distress as first differences that varied between T- and TH-groups and with tinnitus duration. The scores of the G-H-S questionnaire were about twice as high for the TH-group than for the T-group (Figure 3). This means that tinnitus patients who also suffer from hyperacusis are usually much more distressed by their tinnitus than those without hyperacusis, as also observed in previous studies (43, 46).

Although the G-H-S scores in neither group changed in levels over the duration of tinnitus (Figures 3C,F), the intensity of tinnitus, that is, the loudness in dB HL, *decreased* significantly over time (Figure 3B), but *increased* in the TH-group (Figure 3E).

This means that in patients with both tinnitus and hyperacusis, the tinnitus loudness in the first 5 years is about half that of the subjects with tinnitus alone, but when the duration is more than 5 years, the loudness is the same in both groups. It also means that in the T-group, a confounding factor is expected to contribute to the ongoing distress levels, reflected in steadily elevated G-H-S scores over the duration of tinnitus, although in this group the tinnitus level declined (Figure 3B). We here suggest that the confounding factor in the T-group is a gradual increase in hyperacusis characteristics. Before we discuss this aspect in more detail, it is important to note that two recent trials also investigated the transition of tinnitus from acute to chronic over a period of 6 months (29, 30) and found that the average tinnitus distress moderately improved over time. Neither of these studies, however, analyzed tinnitus over longer durations or separated its features from the co-occurrence of hyperacusis.

### A Risk of Tinnitus Burden Through the Co-occurrence of Hyperacusis Increases Over Time

As mentioned above, in the T-group, constant annoyance and distress, evident in total G-H-S questionaire scores as a function of tinnitus duration (Figure 3C), was seen despite a parallel and on-going decline in tinnitus intensity, particularly for more than 5 years (Figure 3B). This is best explained through a parallel occurrence of an increased distress to low sound levels as identified in this group through increasing HKI scores over tinnitus duration (Figure 4A). Also within the same time scale of tinnitus durations of > 5 years, an increase in bilateral tinnitus was most obvious (Figure 5A). Finally, also within this time scale, in the T-group altered ABR wave III response characteristics, as typically noticed only for the TH-group, were observed (Figures 6A, 7D). Overall, this parallel occurrence of changes in the T-group may reveal an increased probability of exhibiting a co-occurrence of hyperacusis after > 5 years of tinnitus duration.

This hypothesis is strengthend by the following observations: **(i)** in the TH-group, the co-occurrence of hyperacusis was characterized right from the beginning through an enhanced annoyance and distress level shown in higher G-H-S questionaire thresholds (Figure 3F). This feature was linked to nearly constant hyperacusis scores (Figures 4C,D) and to an increased tinnitus loudness over time (Figure 3E), indicating that with the co-occurrence of hyperacusis, the tinnitus becomes louder and more annoying; **(ii)** the bilateral experience of tinnitus in the TH-group from the beginning (Figure 5B), together with the higher G-H-S and HKI score thresholds in this group, also from the beginning (Figure 3F) strengthen the concept that bilateral tinnitus is probably linked to the enhanced annoyance of the TH-group. Thus, increased bilateral tinnitus in the T-group with > 5 years of tinnitus duration (Figure 5A) may be associated with the increased HKI scores observed in this group > 5 years of tinnitus duration; **(iii)** The shift of unilateral- to nearly complete bilateral tinnitus sensation in the T-group, most evident from > 5 years (Figure 5A), also coincided with the appearance of the ABR wave III enhancement that is particularly evident > 5 years (Figure 6A, see next chapter). Enhanced ABR wave III amplitudes are a suggested feature of the co-occurrence of hyperacusis in TH-groups (see below), while an increase in bilateral tinnitus and a worse scoring of tinnitus annoyance was previously also found in tinnitus subjects with the co-occurrence of hyperacusis (46). To date, no study has compared psychometric and psychoacoustic differences using detailed audiometry.

### A Risk of Tinnitus Burden Through the Co-occurrence of Hyperacusis Can Be Distinguished Through Objective Differences in ABR Wave Responses

In the present study, profound differences between T- and TH-groups in sound-induced ABR responses were found that support the notion that in future studies T- and TH-groups may be distinguished through reduced and prolonged ABR wave V and reduced ABR wave V/I ratio as a main feature of the T-group (Figures 6A,B, 7A) and enhanced and shortened ABR wave III/V and enhanced ABR wave III/I ratio (Figures 6C,D, 7D) as a characteristic feature of the TH-group (Figures 6A,B). Both characteristics persisted over tinnitus durations of years and were even enhanced in the TH-group over time (Figure 7F). This supports the notion that not tinnitus *per se*, but tinnitus with the co-occurrence of hyperacusis is the hearing disorder that most needs therapeutic intervention in future studies.

Overall, an increased HKI score in the T-group over time (Figures 4A,B), increased tinnitus bilaterality in the T-group over time (Figure 5A), enhanced ABR wave III response characteristics to high-intense sound stimuli that gradually develop in the T-group > 5 years of tinnitus duration (Figures 6A, 7E), and that revealed a greatly augmented risk of the co-occurrence of hyperacusis with increasing tinnitus duration.

### Differences in Neural Correlates Behind T-Group and TH-Group Phenotype Differences

What underlies the reduced and delayed ABR wave V responses in the T-group and enhanced and shortened ABR wave III and wave V response in the TH-group? Here, we may reconsider an ongoing discussion as to whether neural gain (ABR wave V/I ratio) is a marker of tinnitus or not (27, 47). To date, numerous studies have suggested that tinnitus is the result of homeostatic increases in central neural gain [see reviews: (12, 13, 15, 17, 19–22)]. Other studies, including the present findings, proposed that tinnitus is not related to increased central gain (24, 27, 48), but instead occurs when auditory input falls short of the critical firing rate of auditory fibers for increasing neural gain, so that stimulus-evoked responses are diminished in the ascending auditory pathway (23, 24, 48–50). A critical reduction of fast (high spontaneous rate, high-SR) auditory processing has been suggested to be linked to the reduced and delayed late ABR wave V in tinnitus, as diminished fast (high-SR) auditory fiber activity might reduce contrast-amplification circuits that are essential to amplify relevant, and ignore irrelevant, stimuli (47). This implies that tinnitus is brain noise that is ‘heard’ at frequencies of diminished fast (high-SR) auditory processing (47). Disrupted fast (high-SR) auditory processing as a correlate of tinnitus (23, 24, 51) was also suggested to impair fast communication between auditory-specific and fronto-striatal regions and would, therefore, diminish memory-dependent adjustment of stimulus-evoked responses following, for example, acoustic trauma. In the meantime various studies have directly or indirectly supported the notion that tinnitus may be more associated with a reduced neural gain than an enhanced neural gain. This was shown through reduced and delayed late ABR wave V in subacute tinnitus groups (28), reduced sound-evoked BOLD fMRI activity in the auditory cortex (23, 52), reduced functional connectivity observed during sound-evoked activity (39, 53), reduced resting-state functional r-fcMRI connectivity between auditory-specific brain regions and fronto-striatal regions (23, 54) and reductions in the selectivity of frequency tuning that start at the level of the medial geniculate body and continue in the auditory cortex in tinnitus groups (25).

In contrast to the reduced and prolonged ABR wave V responses in the T-group, the TH-group exhibited - as a most characteristic feature - an enhanced and shorter latency wave V and wave III, that even increased further over the duration of tinnitus (Figures 6C,D, 7F). Although to date the neural correlate of hyperacusis is elusive, some studies suggested either an unspecific over-activation of type-II cochlear afferents at the level of OHCs that is triggered by noxious loud sound levels (55, 56) or an over-activation of the auditory brainstem medial olivocochlear (**MOC**) system (57, 58) to be possibly linked with hyperacusis. Considering the first view, it is important to note that an over-activation of type-II cochlear afferents would activate the posteroventral cochlear nucleus PVCN neurons (59) that project to the contra-lateral MOC neurons located in the SOC complex (60). As a consequence, excessive MOC efferent activity would diminish OHC baseline motile responses and inner hair cell output (58). In the view of previous (47, 51) and present findings, this might further accelerate a critical reduction of fast auditory processing in possibly deafferented cochlear regions in the non-tinnitus side and thereby contribute that over time uni-lateral tinnitus becomes bilateral in the T-group (Figure 5). On the other side the same event of diminished OHCs baseline motile response following excessive MOC efferent activity could trigger in other frequency regions compensating enhanced sound-induced brainstem activity (58). Indeed, hyperactivity of MOC neurons and auditory brainstem regions following acoustic trauma has been observed in previous studies (14, 61–65) and through such an overactive response suggested to contribute to a lowering of loudness tolerance (58). As the MOC neurons are located in the SOC complex (60), the cellular generators of ABR wave III (66), the elevated and shortened ABR wave III and V amplitudes here observed in response to high level sound in the TH-group (Figures 6, 7) could be explained through such an event.

As suggested above, as a most striking finding of the present study, we here describe that these events (reduced ABR wave V and enhanced ABR wave III) can co-occur and do so in the T-group over the duration of tinnitus > 5 years, when also other hyperacusis characteristics progress. This suggests that objective markers for either tinnitus or hyperacusis may have indeed been identified. Keeping this in mind, numerous previously controversial findings of either reduced or enhanced central auditory responses in tinnitus (27, 47, 67) may find a rational explanation. Also, previous studies that included tinnitus patients with unilateral and bilateral tinnitus and only found tinnitus characteristics in the sub-chronical group (28) may reconsider the findings in the context of the present study. Moreover, differences for age, tinnitus laterality, and tinnitus pitch, previously reported between tinnitus groups with and without hyperacusis (43, 46) may be reconsidered, in that bilateral tinnitus may progress in the tinnitus group over time, as suggested here. Finally, previous suggestions that tinnitus characteristics were not related to hyperacusis (43) may reassess this in view of a possible progressive co-occurrence of tinnitus and hyperacusis over time.

## Conclusion

We identified a reduced and prolonged ABR wave V with developing additively enhanced ABR wave III responses, increased hyperacusis scores, and increased bilaterality, as common features of the T-group that develop over time, a worsening of the tinnitus pathology that may require more medical care than tinnitus alone. Moreover, the different features of tinnitus with and without hyperacusis identified here, may have contributed to currently ongoing controversial views on the neural basis of tinnitus that exist since decades. On the basis of this finding, we urgently recommend changing medical practice toward a sub-classification of tinnitus with and without hyperacusis with a specific emphasis on tinnitus duration. With the objective tools presented here, mutual efforts and harmonized methods may progress to find urgently needed, personalized therapies for tinnitus in ENT clinics.

## Data Availability Statement

The original contributions presented in the study are included in the article/Supplementary Material, further inquiries can be directed to the corresponding author/s.

## Ethics Statement

The studies involving human participants were reviewed and approved by Ethics Committee of the University of Tübingen. The patients/participants provided their written informed consent to participate in this study.

## Author Contributions

MK, LR, and SW: designed research. FR, BH, and JW: conducting experiments. FR, LR, BH, PH, JW, JS, and WS: analyzed data. SW and LR: wrote the first draft of the paper. MK, WS, UK, BH, JW, JS, HS, and RMA: edited the paper. MK, LR, and SW: wrote the paper. All authors contributed to the article and approved the submitted version.

## Conflict of Interest

The authors declare that the research was conducted in the absence of any commercial or financial relationships that could be construed as a potential conflict of interest.

## References

[1] Friberg E Gustafsson K Alexanderson K. Hearing difficulties, ear-related diagnoses and sickness absence or disability pension–a systematic literature review. BMC Public Health. (2012) 12:772. 10.1186/1471-2458-12-772 22966953 PMC3560197

[2] Baguley DM. Hyperacusis. J R Soc Med. (2003) 96:582–5. 10.1258/jrsm.96.12.582 14645606 PMC539655

[3] Hebert S Paiement P Lupien SJ. A physiological correlate for the intolerance to both internal and external sounds. Hear Res. (2004) 190:1–9. 10.1016/S0378-5955(04)00021-8 15051125

[4] Andersson G Lindvall N Hursti T Carlbring P. Hypersensitivity to sound (hyperacusis): a prevalence study conducted via the Internet and post. Int J Audiol. (2002) 41:545–54. 10.3109/14992020209056075 12477175

[5] Gu JW Halpin CF Nam EC Levine RA Melcher JR. Tinnitus, diminished sound-level tolerance, and elevated auditory activity in humans with clinically normal hearing sensitivity. J Neurophysiol. (2010) 104:3361–70. 10.1152/jn.00226.2010 20881196 PMC3007631

[6] McCormack A Edmondson-Jones M Somerset S Hall D. A systematic review of the reporting of tinnitus prevalence and severity. Hear Res. (2016) 337:70–9. 10.1016/j.heares.2016.05.009 27246985

[7] Aazh H McFerran D Salvi R Prasher D Jastreboff M Jastreboff P. Insights from the First International Conference on Hyperacusis: causes, evaluation, diagnosis and treatment. Noise Health. (2014) 16:123–6. 10.4103/1463-1741.132100 24804717

[8] Chen Z Yuan W. Central plasticity and dysfunction elicited by aural deprivation in the critical period. Front Neural Circuits. (2015) 9:26. 10.3389/fncir.2015.00026 26082685 PMC4451366

[9] Moller AR Salvi R De Ridder D Kleinjung T Vanneste S. Pathology of Tinnitus and Hyperacusis-Clinical Implications. Biomed Res Int. (2015) 2015:608437. 10.1155/2015/608437 26587541 PMC4637452

[10] Chen YC Xia W Chen H Feng Y Xu JJ Gu JP. Tinnitus distress is linked to enhanced resting-state functional connectivity from the limbic system to the auditory cortex. Hum Brain Mapp. (2017) 38:2384–97. 10.1002/hbm.23525 28112466 PMC6866871

[11] Aazh H Salvi R. The Relationship between Severity of Hearing Loss and Subjective Tinnitus Loudness among Patients Seen in a Specialist Tinnitus and Hyperacusis Therapy Clinic in UK. J Am Acad Audiol. (2019) 30:712–9. 10.3766/jaaa.17144 30403955

[12] Norena AJ. An integrative model of tinnitus based on a central gain controlling neural sensitivity. Neurosci Biobehav Rev. (2011) 35:1089–109. 10.1016/j.neubiorev.2010.11.003 21094182

[13] Schaette R McAlpine D. Tinnitus with a normal audiogram: physiological evidence for hidden hearing loss and computational model. J Neurosci. (2011) 31:13452–7. 10.1523/JNEUROSCI.2156-11.2011 21940438 PMC6623281

[14] Yang S Weiner BD Zhang LS Cho SJ Bao S. Homeostatic plasticity drives tinnitus perception in an animal model. Proc Natl Acad Sci U S A. (2011) 108:14974–9. 10.1073/pnas.1107998108 21896771 PMC3169130

[15] Schaette R Kempter R. Computational models of neurophysiological correlates of tinnitus. Front Syst Neurosci. (2012) 6:34. 10.3389/fnsys.2012.00034 22586377 PMC3347476

[16] Yang S Bao S. Homeostatic mechanisms and treatment of tinnitus. Restor Neurol Neurosci. (2013) 31:99–108. 10.3233/RNN-120248 23435453

[17] Auerbach BD Rodrigues PV Salvi RJ. Central gain control in tinnitus and hyperacusis. Front Neurol. (2014) 5:206. 10.3389/fneur.2014.00206 25386157 PMC4208401

[18] Noreña AJ. Revisiting the cochlear and central mechanisms of tinnitus and therapeutic approaches. Audiol Neurootol. (2015) 20 (Suppl 1):53–9. 10.1159/000380749 25997584

[19] Sedley W Friston KJ Gander PE Kumar S Griffiths TD. An Integrative Tinnitus Model Based on Sensory Precision. Trends Neurosci. (2016) 39:799–812. 10.1016/j.tins.2016.10.004 27871729 PMC5152595

[20] Shore SE Roberts LE Langguth B. Maladaptive plasticity in tinnitus–triggers, mechanisms and treatment. Nat Rev Neurol. (2016) 12:150–60. 10.1038/nrneurol.2016.12 26868680 PMC4895692

[21] Marks KL Martel DT Wu C Basura GJ Roberts LE Schvartz-Leyzac KC. Auditory-somatosensory bimodal stimulation desynchronizes brain circuitry to reduce tinnitus in guinea pigs and humans. Sci Transl Med. (2018) 10. 10.1126/scitranslmed.aal3175 29298868 PMC5863907

[22] Roberts LE Salvi R. Overview: Hearing loss, tinnitus, hyperacusis, and the role of central gain. Neuroscience. (2019). 10.1016/j.neuroscience.2019.03.021 30885639

[23] Hofmeier B Wolpert S Aldamer ES Walter M Thiericke J Braun C. Reduced sound-evoked and resting-state BOLD fMRI connectivity in tinnitus. Neuroimage Clin. (2018) 20:637–49. 10.1016/j.nicl.2018.08.029 30202725 PMC6128096

[24] Möhrle D Hofmeier B Amend M Wolpert S Ni K Bing D. Enhanced Central Neural Gain Compensates Acoustic Trauma-induced Cochlear Impairment, but Unlikely Correlates with Tinnitus and Hyperacusis. Neuroscience. (2019) 407:146–69. 10.1016/j.neuroscience.2018.12.038 30599268

[25] Berlot E Arts R Smit J George E Gulban OF Moerel M. A 7 Tesla fMRI investigation of human tinnitus percept in cortical and subcortical auditory areas. Neuroimage Clin. (2020) 25:102166. 10.1016/j.nicl.2020.102166 31958686 PMC6970183

[26] Kleinjung T Langguth B. Avenue for Future Tinnitus Treatments. Otolaryngol Clin North Am. (2020) 53:667–83. 10.1016/j.otc.2020.03.013 32381341

[27] Sedley W. Tinnitus: does gain explain? Neuroscience. (2019) 407:213–28. 10.1016/j.neuroscience.2019.01.027 30690137

[28] Joo JW Jeong YJ Han MS Chang YS Rah YC Choi J. Analysis of auditory brainstem response change, according to tinnitus duration, in patients with tinnitus with normal hearing. J Int Adv Otol. (2020) 16:190–6. 10.5152/iao.2020.7951 32784156 PMC7419086

[29] Wallhausser-Franke E D'Amelio R Glauner A Delb W Servais JJ Hormann K. Transition from acute to chronic tinnitus: predictors for the development of chronic distressing tinnitus. Front Neurol. (2017) 8:605. 10.3389/fneur.2017.00605 29209267 PMC5701924

[30] Vielsmeier V Santiago Stiel R Kwok P Langguth B Schecklmann M. From acute to chronic tinnitus: pilot data on predictors and progression. Front Neurol. (2020) 11:997. 10.3389/fneur.2020.00997 33041971 PMC7516990

[31] Schecklmann M Landgrebe M Poeppl TB Kreuzer P Manner P Marienhagen J. Neural correlates of tinnitus duration and distress: a positron emission tomography study. Hum Brain Mapp. (2013) 34:233–40. 10.1002/hbm.21426 22021023 PMC6870498

[32] Fischer A. Hyperakusis: Neues Screening-instrument vorgestellt. HNO Nachrichten. (2013) 43:38–38. 10.1007/s00060-013-0111-x

[33] Goebel GA Schöffel J Bläsing L. “Das Hyperakusis-Inventar (HKI): Ein valides Screeninginstrument zur Erfassung der Hyperakusisbelastung unter Berücksichtigung von Phonophobie, Rekruitment und Schwerhörigkeit”, in: 84. Jahresversammlung der Deutschen Gesellschaft für Hals-Nasen-Ohren-Heilkunde, Kopf- und Hals-Chirurgie. (ed.) Goebel G.A.B. Schöffel J. Bläsing L.) (2013).

[34] Hiller W Goebel G Rief W. Reliability of self-rated tinnitus distress and association with psychological symptom patterns. Br J Clin Psychol. (1994) 33 (Pt 2):231–9. 8038742 10.1111/j.2044-8260.1994.tb01117.x

[35] Wu V Cooke B Eitutis S Simpson MTW Beyea JA. Approach to tinnitus management. Can Fam Physician. (2018) 64:491–5. 30002023 PMC6042678

[36] Dallos P Harris D. Properties of auditory nerve responses in absence of outer hair cells. J Neurophysiol. (1978) 41:365–83. 650272 10.1152/jn.1978.41.2.365

[37] He DZ Evans BN Dallos P. First appearance and development of electromotility in neonatal gerbil outer hair cells. Hear Res. (1994) 78:77–90. 10.1016/0378-5955(94)90046-9 7961180

[38] Geven LI de Kleine E Free RH van Dijk P. Contralateral suppression of otoacoustic emissions in tinnitus patients. Otol Neurotol. (2011) 32:315–21. 10.1097/MAO.0b013e3181fcf180 20962699

[39] Boyen K de Kleine E van Dijk P Langers DR. Tinnitus-related dissociation between cortical and subcortical neural activity in humans with mild to moderate sensorineural hearing loss. Hear Res. (2014) 312:48–59. 10.1016/j.heares.2014.03.001 24631963

[40] Gilles A Schlee W Rabau S Wouters K Fransen E Van de Heyning P. Decreased Speech-In-Noise Understanding in Young Adults with Tinnitus. Front Neurosci. (2016) 10:288. 10.3389/fnins.2016.00288 27445661 PMC4923253

[41] Guest H Munro KJ Prendergast G Howe S Plack CJ. Tinnitus with a normal audiogram: Relation to noise exposure but no evidence for cochlear synaptopathy. Hear Res. (2017) 344:265–74. 10.1016/j.heares.2016.12.002 27964937 PMC5256478

[42] Hebert S Fournier P Norena A. The auditory sensitivity is increased in tinnitus ears. J Neurosci. (2013) 33:2356–64. 10.1523/JNEUROSCI.3461-12.2013 23392665 PMC6619157

[43] Schecklmann M Landgrebe M Langguth B Group TRIDS. Phenotypic characteristics of hyperacusis in tinnitus. PLoS ONE. (2014) 9:e86944. 10.1371/journal.pone.0086944 24498000 PMC3908961

[44] Gates GA Mills JH. Presbycusis. Lancet. (2005) 366:1111–20. 10.1016/S0140-6736(05)67423-5 16182900

[45] Liberman MC. Noise-induced and age-related hearing loss: new perspectives and potential therapies. F1000Res. (2017) 6:927. 10.12688/f1000research.11310.1 28690836 PMC5482333

[46] Ralli M Salvi RJ Greco A Turchetta R De Virgilio A Altissimi G. Characteristics of somatic tinnitus patients with and without hyperacusis. PLoS One. (2017) 12:e0188255. 10.1371/journal.pone.0188255 29161302 PMC5697853

[47] Knipper M van Dijk P Schulze H Mazurek B Krauss P Scheper V. The neural bases of tinnitus: lessons from deafness and cochlear implants. J Neurosci. (2020) 40:7190–202. 10.1523/JNEUROSCI.1314-19.2020 32938634 PMC7534911

[48] Zeng FG. An active loudness model suggesting tinnitus as increased central noise and hyperacusis as increased nonlinear gain. Hear Res. (2013) 295:172–9. 10.1016/j.heares.2012.05.009 22641191 PMC3593089

[49] Rüttiger L Singer W Panford-Walsh R Matsumoto M Lee SC Zuccotti A. The reduced cochlear output and the failure to adapt the central auditory response causes tinnitus in noise exposed rats. PLoS ONE. (2013) 8:e57247. 10.1371/journal.pone.0057247 23516401 PMC3596376

[50] Singer W Zuccotti A Jaumann M Lee SC Panford-Walsh R Xiong H. Noise-induced inner hair cell ribbon loss disturbs central arc mobilization: a novel molecular paradigm for understanding tinnitus. Mol Neurobiol. (2013) 47:261–79. 10.1007/s12035-012-8372-8 23154938

[51] Knipper M Van Dijk P Nunes I Rüttiger L Zimmermann U. Advances in the neurobiology of hearing disorders: recent developments regarding the basis of tinnitus and hyperacusis. Prog Neurobiol. (2013) 111:17–33. 10.1016/j.pneurobio.2013.08.002 24012803

[52] Koops EA Lanting CP Renken RJ Van Dijk P. Cortical tonotopic maps changes in humans are larger in hearing loss than in additional tinnitus. J Neurosci. (2020) 40:3178–85. 10.1523/JNEUROSCI.2083-19.2020 32193229 PMC7159897

[53] Lanting CP de Kleine E Langers DR van Dijk P. Unilateral tinnitus: changes in connectivity and response lateralization measured with FMRI. PLoS ONE. (2014) 9:e110704. 10.1371/journal.pone.0110704 25329557 PMC4203817

[54] Leaver AM Turesky TK Seydell-Greenwald A Morgan S Kim HJ Rauschecker JP. Intrinsic network activity in tinnitus investigated using functional MRI. Hum Brain Mapp. (2016) 37:2717–35. 10.1002/hbm.23204 27091485 PMC4945432

[55] Flores EN Duggan A Madathany T Hogan AK Marquez FG Kumar G. A non-canonical pathway from cochlea to brain signals tissue-damaging noise. Curr Biol. (2015) 25:606–12. 10.1016/j.cub.2015.01.009 25639244 PMC4348215

[56] Liu C Glowatzki E Fuchs PA. Unmyelinated type II afferent neurons report cochlear damage. Proc Natl Acad Sci USA. (2015) 112:14723–7. 10.1073/pnas.1515228112 26553995 PMC4664349

[57] Knudson IM Shera CA Melcher JR. Increased contralateral suppression of otoacoustic emissions indicates a hyperresponsive medial olivocochlear system in humans with tinnitus and hyperacusis. J Neurophysiol. (2014) 112:3197–208. 10.1152/jn.00576.2014 25231612 PMC4269714

[58] Sturm JJ Weisz CJ. Hyperactivity in the medial olivocochlear efferent system is a common feature of tinnitus and hyperacusis in humans. J Neurophysiol. (2015) 114:2551–4. 10.1152/jn.00948.2014 25695650 PMC4630185

[59] Morgan YV Ryugo DK Brown MC. Central trajectories of type II (thin) fibers of the auditory nerve in cats. Hear Res. (1994) 79:74–82. 10.1016/0378-5955(94)90128-7 7806486

[60] Darrow KN Benson TE Brown MC. Planar multipolar cells in the cochlear nucleus project to medial olivocochlear neurons in mouse. J Comp Neurol. (2012) 520:1365–75. 10.1002/cne.22797 22101968 PMC3514887

[61] Salvi RJ Wang J Ding D. Auditory plasticity and hyperactivity following cochlear damage. Hear Res. (2000) 147:261–74. 10.1016/s0378-5955(00)00136-2 10962190

[62] Cai S Ma WL Young ED. Encoding intensity in ventral cochlear nucleus following acoustic trauma: implications for loudness recruitment. J Assoc Res Otolaryngol. (2009) 10:5–22. 10.1007/s10162-008-0142-y 18855070 PMC2644394

[63] Middleton JW Kiritani T Pedersen C Turner JG Shepherd GM Tzounopoulos T. Mice with behavioral evidence of tinnitus exhibit dorsal cochlear nucleus hyperactivity because of decreased GABAergic inhibition. Proc Natl Acad Sci U S A. (2011) 108:7601–6. 10.1073/pnas.1100223108 21502491 PMC3088638

[64] Vogler DP Robertson D Mulders WH. Hyperactivity in the ventral cochlear nucleus after cochlear trauma. J Neurosci. (2011) 31:6639–45. 10.1523/JNEUROSCI.6538-10.2011 21543592 PMC6632868

[65] Groschel M Ryll J Gotze R Ernst A Basta D. Acute and long-term effects of noise exposure on the neuronal spontaneous activity in cochlear nucleus and inferior colliculus brain slices. Biomed Res Int. (2014) 2014:909260. 10.1155/2014/909260 25110707 PMC4119618

[66] Melcher JR Kiang NY. Generators of the brainstem auditory evoked potential in cat. III: Identified cell populations Hear Res. (1996) 93:52–71. 8735068 10.1016/0378-5955(95)00200-6

[67] Sheppard A Stocking C Ralli M Salvi R. A review of auditory gain, low-level noise and sound therapy for tinnitus and hyperacusis. Int J Audiol. (2020) 59:5–15. 10.1080/14992027.2019.1660812 31498009

