# Supplementary material for: Co-occurrence of Hyperacusis Accelerates With Tinnitus Burden Over Time and Requires Medical Care
Source: Front Neurol. 2021 Mar 18;12:627522. doi: 10.3389/fneur.2021.627522 (PMC8012887; doi:10.3389/fneur.2021.627522)
Supplement: Supplementary file 1 [file Data_Sheet_1.docx]

**Supplementary Table 1:** Study participants and demographic data

| **Control** | | | | **Tinnitus <1yr** | | | | | **Tinnitus+Hyperacusis <1yr** | | | | |
| --- | --- | --- | --- | --- | --- | --- | --- | --- | --- | --- | --- | --- | --- |
| *No* | *age* | *sex* | *hand* | *No* | *age* | *sex* | *hand* | *duration* | *No* | *age* | *sex* | *hand* | *duration* |
| C1 | 27 | m | r | T1 | 33 | m | r | 0.5 | TH1 | 24 | f | r | 0.25 |
| C2 | 41 | f | l | T2 | 27 | f | r | 0.5 | TH2 | 21 | f | r | 0.5 |
| C3 | 22 | m | r | T3 | 61 | m | r | 0.5 | TH3 | 49 | m | l | 0.5 |
| C4 | 27 | m | l | T4 | 25 | m | r | 0.6 | TH4 | 27 | m | r | 0.5 |
| C5 | 19 | m | r | T5 | 34 | m | l | 0.8 | TH5 | 21 | f | l | 0.8 |
| C6 | 18 | m | r | T6 | 31 | m | r | 1 | TH6 | 21 | f | r | 1 |
| C7 | 39 | m | r |  |  |  |  |  | TH7 | 33 | m | r | 1 |
| C8 | 26 | m | r |  |  |  |  |  | TH8 | 36 | f | r | 1 |
| C9 | 24 | f | r |  | | | | |  | | | | |
| C10 | 21 | f | r | **Tinnitus 1-5yr** | | | | | **Tinnitus+Hyperacusis 1-5 yr** | | | | |
| C11 | 28 | m | r | *No* | *age* | *f/m* | *hand* | *duration* | *No* | *age* | *sex* | *hand* | *duration* |
| C12 | 31 | m | r | T7 | 26 | m | r | 1.5 | TH9 | 26 | f | r | 1.2 |
| C13 | 30 | f | r | T8 | 36 | m | r | 1.5 | TH10 | 20 | m | l | 1.5 |
| C14 | 19 | m | r | T9 | 50 | m | r | 1.6 | TH11 | 22 | f | r | 2.8 |
| C15 | 26 | m | r | T10 | 20 | f | r | 2 | TH12 | 29 | m | r | 3.5 |
| C16 | 26 | f | r | T11 | 31 | f | l | 2 |  |  |  |  |  |
| C17 | 27 | m | r | T12 | 20 | f | r | 2.5 |  |  |  |  |  |
| C18 | 27 | m | r | T13 | 56 | f | r | 2.5 |  |  |  |  |  |
| C19 | 24 | f | r | T14 | 24 | m | r | 3 |  |  |  |  |  |
| C20 | 26 | m | r | T15 | 44 | f | r | 3 |  |  |  |  |  |
| C21 | 45 | f | r |  | | | | |  | | | | |
| C22 | 27 | f | r | **Tinnitus 5-10 years** | | | | | **Tinnitus+hyperacusis 5-10 years** | | | | |
| C23 | 26 | f | r | *No* | *age* | *f/m* | *hand* | *duration* | *No* | *age* | *sex* | *hand* | *duration* |
| C24 | 32 | f | r | T16 | 25 | f | r | 5 | TH14 | 24 | m | r | 5 |
| C25 | 21 | f | r | T17 | 44 | m | r | 6 | TH15 | 34 | f | r | 5 |
| C26 | 23 | f | r | T18 | 21 | f | r | 6 | TH16 | 24 | f | r | 6 |
| C27 | 18 | f | r | T19 | 23 | m | l | 6 | TH17 | 23 | f | r | 6 |
| C28 | 20 | f | r | T20 | 21 | m | r | 7 | TH14 | 30 | f | r | 8 |
| C29 | 20 | f | r | T21 | 26 | m | r | 7 |  |  |  |  |  |
| C30 | 24 | m | l | T22 | 27 | m | r | 7 |  |  |  |  |  |
| C31 | 26 | m | r | T23 | 49 | m | r | 7 |  |  |  |  |  |
| C32 | 22 | f | r | T24 | 25 | f | r | 8 |  |  |  |  |  |
| C33 | 26 | f | r |  | | | | |  | | | | |
| C34 | 25 | f | l | **Tinnitus >10yr** | | | | | **Tinnitus+Hyperacusis >10yr** | | | | |
| C35 | 31 | m | r | *No* | *age* | *sex* | *hand* | *duration* | *No* | *age* | *sex* | *hand* | *duration* |
| C36 | 28 | f | r | T25 | 29 | m | r | 10 | TH18 | 24 | f | r | 12 |
| C37 | 22 | m | r | T26 | 36 | m | l | 11 | TH19 | 23 | f | r | 13 |
| C38 | 35 | f | r | T27 | 29 | m | r | 14 | TH20 | 28 | f | r | 18 |
| C39 | 30 | f | r | T28 | 26 | m | l | 15 |  |  |  |  |  |
| C40 | 27 | m | r | T29 | 29 | m | r | 15 |  |  |  |  |  |
| C41 | 24 | f | r | T30 | 61 | f | r | 15 |  |  |  |  |  |
| C42 | 27 | f | r | T31 | 35 | f | r | 19 |  |  |  |  |  |
| C43 | 33 | m | r | T32 | 27 | m | r | 21 |  |  |  |  |  |
|  |  |  |  | T33 | 26 | m | r | 22 |  |  |  |  |  |

C#: participant of Control group
T#: Participant number of Tinnitus group, TH#: participant number of Tinnitus+Hyperacusis group
f: female, m: male, hand: handedness, l: left-handed, r: right-handed, duration: time of tinnitus persistence

**Supplementary Table 2:** Tinnitus description and evaluation

| Study group | GHS | HKI | Hz (right) | dB (right) | Hz (left) | dB (left) |
| --- | --- | --- | --- | --- | --- | --- |
| T1 | 12 | 7 | 10.000 | 28 | ‐‐‐ | ‐‐‐ |
| T2 | 5 | 4 | 1500 | 15 | ‐‐‐ | ‐‐‐ |
| T3 | 10 | 7 | 4000 | 23 | ‐‐‐ | ‐‐‐ |
| T4 | 8 | 3 | 4000 | 24 | ‐‐‐ | ‐‐‐ |
| T5 | 48 | 5 | ‐‐‐ | ‐‐‐ | 10.000 | 31 |
| T6 | 17 | 5 | 6000 | 30 | 6000 | 70 |
| T7 | 13 | 7 | 8000 | 14 | 8000 | 18 |
| T8 | 9 | 3 | ‐‐‐ | ‐‐‐ | 8000 | 8 |
| T9 | 38 | 8 | 6000 | 34 | 6000 | 34 |
| T10 | 26 | 6 | 500 | 16 |  |  |
| T11 | 7 | 7 | 8000 | 31 | ‐‐‐ | ‐‐‐ |
| T12 | 19 | 6 | ‐‐‐ | ‐‐‐ | 500 | 10 |
| T13 | 6 | 3 | 6000 | 19 | 6000 | 38 |
| T14 | 4 | 1 | 8000 | 57 | ‐‐‐ | ‐‐‐ |
| T15 | 6 | 6 | 125 | 11 | ‐‐‐ | ‐‐‐ |
| T16 | 7 | 7 | 4000 | 12 | 8000 | 7 |
| T17 | 22 | 4 | ‐‐‐ | ‐‐‐ | 1000 | 13 |
| T18 | 9 | 9 | 6000 | 11 | 8000 | 9 |
| T19 | 7 | 11 | 4000 | 5 | 4000 | 6 |
| T20 | 3 | 3 | 10.000 | 6 | 10.000 | 4 |
| T21 | 24 | 8 | 10.000 | 35 | ‐‐‐ | ‐‐‐ |
| T22 | 10 | 2 | 8000 | 13 | 8000 | 15 |
| T23 | 4 | 6 | 10.000 | 47 | 10.000 | 48 |
| T24 | 10 | 7 | 6000 | 14 | 8000 | 7 |
| T25 | 18 | 9 | 6000 | 18 | 6000 | 14 |
| T26 | 0 | 0 | 8000 | 15 | 8000 | 15 |
| T27 | 16 | 11 | 6000 | 17 | 6000 | 17 |
| T28 | 19 | 9 | 8000 | 15 | 6000 | 3 |
| T29 | 7 | 6 | 3000 | 15 | 3000 | 21 |
| T30 | 44 | 8 | 4000 | 41 | 3000 | 26 |
| T31 | 13 | 11 | 3000 | 23 | 3000 | 26 |
| T32 | 13 | 8 | 10.000 | 13 | 10.000 | 10 |
| T33 | 15 | 5 | 4000 | 5 | 4000 | 1 |
| TH1 | 35 | 12 | ‐‐‐ | ‐‐‐ | 6000 | 20 |
| TH2 | 45 | 14 | 6000 | 9 | 6000 | 10 |
| TH3 | 32 | 15 | 6000 | 10 | 6000 | 18 |
| TH4 | 32 | 18 | 8000 | 30 | 6000 | 9 |
| TH5 | 9 | 12 | ‐‐‐ | ‐‐‐ | 10000 | 5 |
| TH6 | 22 | 15 | 6000 | 2 | 6000 | 1 |
| TH7 | 18 | 15 | 6000 | 9 | 6000 | 25 |
| TH8 | 23 | 25 | 1500 | 10 | 4000 | 12 |
| TH9 | 31 | 12 | 8000 | 8 | 8000 | 8 |
| TH10 | 23 | 17 | ‐‐‐ | ‐‐‐ | 750 | 5 |
| TH11 | 23 | 12 | 4000 | 15 | 4000 | 15 |
| TH12 | 57 | 20 | 10000 | 15 | 10000 | 16 |
| TH14 | 18 | 15 | 1000 | 11 | 8000 | 22 |
| TH15 | 16 | 18 | 10000 | 20 | ‐‐‐ | ‐‐‐ |
| TH16 | 19 | 14 | 1000 | 11 | 750 | 13 |
| TH17 | 53 | 22 | 4000 | 15 | 4000 | 20 |
| TH14 | 28 | 13 | 8000 | 40 | 6000 | 23 |
| TH18 | 31 | 19 | 1000 | 13 | 10000 | 9 |
| TH19 | 27 | 15 | 8000 | 9 | 6000 | 11 |
| TH20 | 30 | 19 | 10000 | 21 | 1000 | 16 |

T##: Participant number of Tinnitus group, TH##: participant number of Tinnitus+Hyperacusis group
GHS: score from Goebel-Hiller tinnitus Questionnaire, ---: inaudible.

**Supplementary Table 3:** Study inclusion/exclusion requirements

| Study inclusion criteria | | | |
| --- | --- | --- | --- |
| Control | | Tinnitus | Tinnitus+Hyperacusis |
| - No tinnitus or hyperacusis suffering at the time of examination or as a medical history | | - History of continuous tinnitus of more than 4 weeks (Tinnitus in one or both sides) - Free from hyperacusis (HKI score ≤11) | - History of continuous tinnitus of more than 4 weeks (Tinnitus in one or both sides) - Suffering from hyperacusis (HKI score >11) |
|  | - PTA with low-grade hearing loss at a hearing threshold up to 40 dB - Clinically free microscopic ear examination and normal eardrum mobility - Age >18 to <70 years - Signed consent form. Written and oral information, which takes place  on request in the native language of the subjects | | |
| Study exclusion criteria for all participants | | | |
| 1. Immune-suppressive drugs (*e.g.* daily cortisone) 2. Pulsatile tinnitus 3. Intermittent non-persistent tinnitus 4. Retro-cochlear hearing loss (as detected by ABR) 5. Hearing aid 6. Vertigo 7. Acoustic trauma 8. Diabetes mellitus type I and type II 9. Pregnancy 10. Drug therapy for tinnitus in the last 4 weeks 11. Ear surgery (*e.g.* tympanoplasty, middle-ear implants) 12. Tinnitus as a secondary symptom underlying another disease (*e.g.* acoustic neuroma or drug tinnitus) 13. Menière disease, endolymphatic hydrops 14. Unilateral or bilateral deafness 15. History of years of noise exposure 16. History of craniocerebral trauma (grade II/III) 17. Cervicogenic or jaw-related tinnitus 18. Conductive hearing with a hearing loss of more than 10 dB at more than 2 frequencies 19. Chronic ear canal or middle-ear infections 20. History of epilepsy suffering, Parkinson's disease and/or dementing illness 21. History of concomitant treatment for neurological and psychiatric disorders (*e.g.* Schizophrenia, depression) 22. Drug or alcohol dependency 23. Renal impairment with an increased creatinine (>160 mol/l = 1.8 mg/dl) 24. Currently in treatment due to a cancerous condition (*e.g.* leukemia) 25. Fear of closed spaces (claustrophobia) 26. Clinical history of cardiovascular disease (severe coronary heart disease) 27. Clinical history restricted temperature sensation and/or increased sensitivity to heating of the body 28. Incapacitated subjects 29. Subjects whom their German is not sufficiently powerful to the instructions to be understood in the context of the study and the questions of the Tinnitus Questionnaire 30. Permanent metal parts in or on the body (pacemakers, artificial heart valves), metal prostheses implanted, magnetic metal parts (screws, plates of operations), spiral, metal fragments/shrapnel, fixed braces, acupuncture needle, insulin pump, intraport, large tattoos) 31. Contrast agent allergy or hypersensitivity to contrast agents 32. Treatments of tinnitus maskers, noiser, hyperbaric oxygen or acupuncture <4 weeks | | | |

**Supplementary Table 4**: Statistical significance of ABR wave amplitude and latency differences.

| **Study Group**  Δ source of variation | 2-way ANOVA | | | pairwise comparisons | | | | |
| --- | --- | --- | --- | --- | --- | --- | --- | --- |
| **Tinnitus** | *wave* | F; *P* | *<1* | | *1-5* | | *5-10* | *>10* |
| ABR amplitude | I | **F (4, 285) = 2.481; *P = 0.0441** |  | |  | |  |  |
| Δ to Control | III | F (4, 291) = 1.354;  P = 0.2500 |  | |  | |  |  |
|  | V | **F (4, 869) = 3.653;**  ****P = 0.0058** |  | | 75dB (t=3.785; ***p=0.0007) | |  | 75dB (t=3.319; **p=0.0028) |
|  | VI | F (4, 268) = 1.314;  P = 0.2651 |  | |  | |  |  |
| ABR latency | I | F (4, 286) = 1.117;  P = 0.3485 |  | |  | |  |  |
| Δ to Control | III | F (4, 292) = 0.826;  P = 0.5095 |  | |  | |  |  |
|  | V | F (4, 869) = 16.28;  ******P < 0.0001** | 75dB (t=2.341; *p=0.0385)  35 dB (t=3.464; **p=0.0017)  25 dB (t=2.396; *p=0.0333) | | 75dB (t=4.116; ***p=0.0002)  65dB (t=2.921; *p=0.0143)  55 dB (t=2.554; *p=0.0438)  45 dB (t=4.057; ***p=0.0002)  35 dB (t=5.25; ****p<0.0001)  25 dB (t=3.782; ***p=0.0005) | | 25 dB (t=2.336; *p=0.0333) | 75dB (t=2.496; *p=0.0377)  45 dB (t=3.428; **p=0.0019)  35 dB (t=3.081; **p=0.0042)  25 dB (t=3.854; ***p=0.0005) |
|  | VI | **F (4, 269) = 3.046;**  ***P = 0.0177** |  | |  | |  |  |
| ABR amplitude | I | **F (3, 120) = 3.640;  *P = 0.0148** |  | | \|---- * t = 2.743 | | ----\| *p = 0.0414 |  |
| Δ duration | III | F (3, 124) = 2.244;  P = 0.0865 |  | |  | |  |  |
|  | V | F (3, 370) = 1.513;  P = 0.2108 |  | |  | |  |  |
|  | VI | F (3, 117) = 0.38;  P = 0.7673 |  | |  | |  |  |
| ABR latency | I | F (3, 121) = 1.325;  P = 0.2694 |  | |  | |  |  |
| Δ duration | III | F (3, 124) = 1.834;  P = 0.1444 |  | |  | |  |  |
|  | V | F (3, 370) = 4.674;  ****P = 0.0032** |  | |  | |  |  |
|  | VI | F (3, 117) = 2.370;  P = 0.0741 |  | |  | |  |  |
| **Tinnitus+Hyperacusis** | *wave* | F; *P* | *<1* | | *1-5* | | *5-10* | *>10* |
| ABR amplitude | I | F (4, 233) = 0.84;  P = 0.5008 |  | |  | |  |  |
| Δ to Control | III | F (4, 238) = 0.657;  P = 0.6225 |  | |  | |  |  |
|  | V | **F (4, 704) = 3.675**  ****P = 0.0057** |  | |  | |  |  |
|  | VI | F (4, 210) = 1.459;  P = 0.2159 |  | |  | |  |  |
| ABR latency | I | **F (4, 233) = 2.383;**  **P = 0.0522** |  | |  | |  |  |
| Δ to Control | III | F (4, 240) = 0.528;  P = 0.7149 |  | |  | |  |  |
|  | V | **F (4, 704) = 3.698;**  ****P = 0.0055** |  | |  | | 25 dB (t=2.998; *p=0,0112) |  |
|  | VI | F (4, 212) = 0.948;  P = 0.4370 |  | |  | |  |  |
| ABR amplitude | I | F (3, 68) = 0.757;  P = 0.5223 |  | |  | |  |  |
| Δ duration | III | F (3, 71) = 07516;  P = 0.5250 |  | |  | |  |  |
|  | V | F (3, 205) = 2.210;  P = 0.0881 |  | |  | |  |  |
|  | VI | F (3, 59) = 1.512;  P = 0.2208 |  | |  | |  |  |
| ABR latency | I | F (3, 68) = 2.266;  P = 0.0886 |  | |  | |  |  |
| Δ duration | III | F (3, 72) = 1.742;  P = 0.1660 |  | |  | |  |  |
|  | V | **F (3, 205) = 3.488;**  ***P = 0.0167** |  | |  | |  |  |
|  | VI | F (3, 60) = 0.7423;  P = 0.5311 |  | |  | |  |  |
| **Study Group**  Δ source of variation |  |  | | | | 2-way ANOVA and pairwise comparison | | |
| **Tinnitus vs. Tinnitus+ Hyperacusis** | *wave* |  | *<1* | | *1-5* | | *5-10* | *>10* |
| ABR amplitude | I |  | F (1, 51) = 3.208;  P = 0.0792 | | F (1, 47) = 2,313;  P = 0.135 | | F (1, 47) = 1,487;  P = 0.2287 | F (1, 43) = 0,2039;  P = 0.6539 |
| Δ T vs T&H | III |  | **F (1, 51) = 5.954;**  ***P = 0.0182** 75dB (t=2.872; *p=0.0118) | | **F (1, 48) = 3,795;**  **P = 0.0573** | | F (1, 52) = 0,1434;  P = 0.7065 | **F (1, 44) = 4,437;**  ***P = 0.0409** 75dB (t=2.422; *p=0.0389) |
|  | V |  | **F(1,153)=14.27;  ***P = 0.0002** 75dB (t=2.672; *p=0.0492) | | **F(1,143)=14.49;  ***P = 0.0002** 75dB (t=4.564; ****p=<0.0001) (0.047) | | **F(1,148)=14.54;  ***P = 0.0002** 75dB (t=3.238; **p=0.0089) | **F(1,131)=37.65;  ****P < 0.0001** 75dB (t=2.77; *p=0.0317)  45dB (t=2.655; *p=0.0352)  25dB (t=2.962; *p=0.0216) |
|  | VI |  | **F (1, 46) = 8.523;**  ***P = 0.0054** 65dB (t=2.424; *p=0.0383) | | F (1, 43) = 2.552;  P = 0.1175 | | F (1, 44) = 2.104;  P = 0.154 | F (1, 43) = 0.2016;  P = 0.6557 |
| ABR latency | I |  | F (1, 51) = 0.172; P = 0.6801 | | **F (1, 47) = 11.17;**  ****P = 0.0016** 65dB (t=1.73; **p=0.0094) | | **F (1, 48) = 5.707;**  ***P = 0.0209** | F (1, 43) = 1.096; P = 0.301 65 dB (t=2.44; *p=0.0374) |
| Δ T vs T&H | III |  | F (1, 52) = 0.065;  P = 0.7997 | | **F (1, 48) = 12.25;**  ****P = 0.001** 75dB (t=2.352; *p=0.0247)  65dB (t=2.597; *p=0.0247) | | **F (1, 52) = 13.23;**  *****P = 0.0006** 75dB (t=2.956; **p=0.0093)  65dB (t=2.187; *p=0.0333) | F (1, 44) = 13.23;  *****P = 0.0007** 65dB (t=3.721; **p=0.0011) |
|  | V |  | **F (1, 153) = 29.26;**  ******P < 0.0001**  45dB (t=2.639; *p=0.0362)  35dB (t=3.788; **p=0.0013)  25dB (t=3.014; *p=0.015) | | **F (1, 143) = 35.12;**  ******P < 0.0001**  35dB (t=3.459; **p=0.0036)  25dB (t=3.512; **p=0.0036) | | **F (1, 148) = 34.99**  ******P < 0.0001**  45dB (t=2.639; **p=0.0024)  35dB (t=3.788; **p=0.0061)  25dB (t=3.014; ***p=0.0001) | **F (1, 131) = 60.97**  ******P < 0.0001** 45dB (t=3.844; ***p=0.0008)  35dB (t=3.91; ***p=0.0007)  25dB (t=4.605; ****p<0.0001) |
|  | VI |  | **F (1, 46) = 10.63;**  ***P = 0.0021** 65dB (t=2.847; *p=0.0131) | | F (1, 44) = 0.417;  P = 0.5216 | | **F (1, 44) = 7.243;**  ***P = 0.01** | F (1, 43) = 0.0277;  P = 0.8685 |

* P < 0.05, ** p < 0.01, *** p < 0.001 **** p<0.0001 in post-hoc Holm-Sidak's multiple comparison test, only p-values < 0.05 are reported.
<1, 1-5, 5-10, >10 time of tinnitus persistence (duration, in years)
(0.000) p values for interaction of factor group and stimulus level from 2-way ANOVA, only p-values < 0.05 are reported.
75, 65, 55, 45, 35, 25 dB nHL stimulus level
|--- * ---| significant difference in post-hoc Holm-Sidak's multiple comparison test between these groups
2-way ANOVA P-values < 0.06 are indicated in **bold** typeface
